# Resolving kinetic intermediates during the regulated assembly and disassembly of fusion pores

**DOI:** 10.1038/s41467-019-14072-7

**Published:** 2020-01-13

**Authors:** Debasis Das, Huan Bao, Kevin C. Courtney, Lanxi Wu, Edwin R. Chapman

**Affiliations:** Department of Neuroscience and Howard Hughes Medical Institute, University of Wisconsin-Madison, 1111 Highland Avenue, Madison, WI 53705 USA

**Keywords:** Exocytosis, Neuroscience, Synaptic transmission, Synaptic vesicle exocytosis

## Abstract

The opening of a fusion pore during exocytosis creates the first aqueous connection between the lumen of a vesicle and the extracellular space. Soluble *N*-ethylmaleimide-sensitive factor attachment protein receptors (SNAREs) mediate the formation of these dynamic structures, and their kinetic transitions are tightly regulated by accessory proteins at the synapse. Here, we utilize two single molecule approaches, nanodisc-based planar bilayer electrophysiology and single-molecule FRET, to address the relationship between SNARE complex assembly and rapid (micro-millisecond) fusion pore transitions, and to define the role of accessory proteins. Synaptotagmin (syt) 1, a major Ca^2+^-sensor for synaptic vesicle exocytosis, drove the formation of an intermediate: committed *trans*-SNARE complexes that form large, stable pores. Once open, these pores could only be closed by the action of the ATPase, NSF. Time-resolved measurements revealed that NSF-mediated pore closure occurred via a complex ‘stuttering’ mechanism. This simplified system thus reveals the dynamic formation and dissolution of fusion pores.

## Abstract (web summary)

SNAREs mediate the formation of a fusion pore during exocytosis which connects the lumen of a vesicle with the extracellular space. Here, authors use single molecule approaches to define the role of synaptotagmin 1 and NSF in synaptic pore formation and dissolution.

## Introduction

The mechanism by which proteins catalyze the fusion of cellular membranes remain a central unanswered question in cell biology. In all eukaryotic cells, the majority of fusion events are mediated by soluble *N*-ethylmaleimide-sensitive factor attachment protein receptor (SNARE) proteins^1,2^. The cytoplasmic domains of vesicular SNAREs (v-SNAREs) bind to cognate domains on target membrane SNAREs (t-SNAREs), forming *trans*-complexes that are necessary and sufficient for fusion^3,4^.

Here we focus on the SNAREs that mediate synaptic vesicle (SV) exocytosis in nerve terminals. A key intermediate in this pathway is called the fusion pore, which represents the first aqueous connection between the lumen of a secretory vesicle and the cell exterior^5^. These are nanometer-scale transient structures, lasting only milliseconds before they either close or dilate as the vesicle membrane collapses into the plasmalemma^5,6^. While their structure remains unknown, recent studies indicate that exocytotic pores are composed of both the transmembrane domains (TMDs) of SNARE proteins and phospholipids^7^. Because they are crucial intermediates, fusion pores are a focal point for the action of regulatory proteins^8^. In nerve terminals, fusion pore opening is thought to be triggered by the binding of Ca^2+^ ions to synaptotagmin 1 (syt1)^9,10^.

Following complete fusion, v- and t-SNAREs lie within the plasma membrane, in *cis*-complexes, and must be disassembled to allow for segregation of SNAREs for future rounds of fusion^11,12^. Disassembly occurs when the soluble factor, α-SNAP, binds *cis*-SNARE complexes and recruits the AAA+ ATPase, NSF; hydrolysis of ATP by NSF drives disassembly. While NSF clearly disassembles *cis*-SNARE complexes, it remains unclear as to whether the *trans*-SNARE complex also serves as a substrate^13,14^. This is a crucial question; if *trans*-SNARE complexes are substrates, then the action of NSF should result in the closure of fusion pores. This would have ramifications in, for example, kiss-and-run exocytosis^15^

The regulated assembly and disassembly of fusion pores have been difficult to study because they are short-lived, so it has been challenging to capture intermediate states. Kinetic analysis of these transitions would provide crucial information concerning the action of regulatory factors that help to assemble, and potentially disassemble, pores. To date, most mechanistic studies of reconstituted fusion machines rely on assays with limited time resolution (i.e., seconds to minutes), thus obscuring dynamics.

To delve into the relationship between the status of *trans*-SNARE complexes and fusion pore dynamics, we developed a nanodisc (ND)–black lipid membrane (BLM) system^16^. *Trans*-SNARE pairing results in the formation of individual recombinant fusion pores that can be studied for extended periods because NDs trap pores in intermediate states. The strength of this approach is that pores can be interrogated electrophysiologically^17,18^, thus affording microsecond time resolution to reveal kinetic intermediates that could not be previously observed in defined systems.

Here we combine ND-BLM experiments and single-molecule fluorescence resonance energy transfer (smFRET) measurements^19^ to determine the impact of key regulatory factors on the structure and kinetic properties of fusion pores and *trans*-SNARE complexes. The first goal was to assess whether Ca^2+^•syt1 directly regulates SNAREs to trigger pore opening. Indeed, Ca^2+^ and syt1 affected not only the occurrence but also the size and kinetic stability of purely recombinant fusion pores. The observation that Ca^2+^•syt1 dilates pores prompted experiments to address the state of the *trans*-SNARE complexes that formed them. These experiments demonstrated that Ca^2+^•syt1 mediates the formation of an intermediate: committed *trans*-SNARE complexes that can only be disassembled by the action of NSF. The time resolution afforded by the ND-BLM system resolved short-lived fusion pore states and indicated that disassembly occurred via a complex kinetic mechanism. These fusion pore intermediates correspond with *trans*-SNARE complex status and reveal coupling of SNARE complex assembly with fusion pore transitions.

## Results

### A Ca^2+^ switch converts syt1 from fusion clamp to activator

**Fig. 1:**
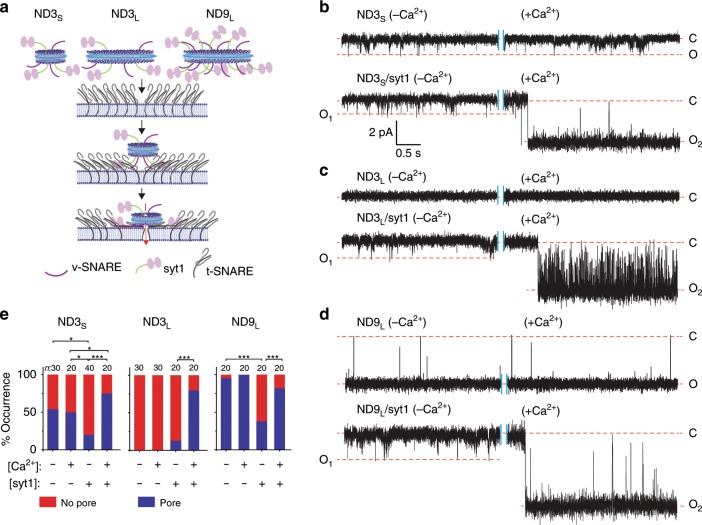
Syt1 regulation of single fusion pores measured via planar lipid bilayer electrophysiology. **a** Illustration of the nanodisc-black lipid membrane (ND-BLM) system, drawn to scale, indicating the different ND preparations used in this study. The number indicates the syb2/syt1 copy number (1:1) per ND; the subscript S stands for small, 13 nm, NDs and the subscript L stands for large, 30 nm, NDs. Traces of single pores with/without syt1 are shown for ND3_S_ (**b**), ND3_L_ (**c**), and ND9_L_ (**d**). In each trace, minus (−) Ca^2+^ contains 1 mM BAPTA (in all bilayer recording experiments in this study) and plus (+) Ca^2+^ contains 500 µM [Ca^2+^]_free_. Closed (C) and open (O) states are shown; the current and time scale, for all traces, is shown in the inset in **b**. O_1_ and O_2_ indicate open state currents obtained before and after addition of Ca^2+^, respectively. **e** Fraction of trials in which a fusion pore was detected, plotted as percentage of occurrence. ND3_S_, ND3_L_, and ND9_L_, in the presence (+) or absence (−) of Ca^2+^ and in the presence (+) and absence (−) of syt1 were compared. Three independent sets of NDs of each type were used, and the total number of measurements obtained under each condition (*n*) is indicated. Pearson’s χ^2^ analysis of pores formed by ND3_S_, ND3_L_, and ND9_L_ was performed; *p < 0.05, ***p < 0.001.

In cells, manipulation of a number of syt isoforms alters the occurrence and properties of individual fusion pores^20–23^, but it was not clear whether these were direct or indirect effects. To address this question, we used our recently described ND-BLM electrophysiology assay^16^ to study pores with microsecond time resolution. We reconstituted full-length syt1, along with syb2, into NDs (Fig. 1a) and t-SNAREs (syntaxin-1A and SNAP-25B heterodimers) into BLMs (Supplementary Fig. 1a); pore formation between the ND and BLM was monitored via the currents that were detected (Fig. 1b). Three kinds of ND preparations were used: ND3_S_, ND3_L_, and ND9_L_, where 3 and 9 refer to the syb2 (and syt1) copy number per ND and subscripts S and L refer to the small (13 nm) and large (30 nm) diameters of the NDs. We first describe representative traces, followed by quantitative analysis of the pores that were formed.

We began with ND3_S_ and confirmed our earlier observation^16^ that these NDs gave rise to pores that remained mostly in the closed state but transiently flickered open to yield small, transient currents; Ca^2+^ had no obvious effect (Fig. 1b). When syt1 was co-reconstituted with syb2 into ND3_S_, the pore remained mostly in the closed state. However, addition of Ca^2+^ (500 µM [Ca^2+^]_free_ in all experiments, unless otherwise indicated) resulted in the formation of large, stable pores (Fig. 1b). We note that the properties of individual pores did not differ at the beginning, middle, or end of a recording, but all pores closed within ~90 min. Once the terminal closure occurred, there were no further openings or flickers. Closure might involve reversion to a hemi-fused state^24^. When syt1 alone was reconstituted (three copies) into NDs (ND0), pores failed to form either in the presence or absence of Ca^2+^ (Supplementary Fig. 1b), confirming that pore formation required *trans*-SNARE pairing. For the Ca^2+^-free conditions, BAPTA was used to chelate any residual Ca^2+^ present in the buffers; in most of our experiments, we subsequently added Ca^2+^ to yield the indicated [Ca^2+^]_free_ (note: we confirmed that BAPTA binds Ca^2+^ with a stoichiometry of 1:1 in Supplementary Fig. 1c).

Next, we examined ND3_L_ (Fig. 1a) but were unable to detect pore formation in the absence of syt1 (Fig. 1c). These finding suggest that v-SNARE density (ND3_S_, 0.022 syb2/nm^2^, ND3_L_: 0.0042 syb2/nm^2^) and not just copy number is a crucial parameter for pore formation. Indeed, a recent modeling study suggested that restricting the mobility of SNAREs, to increase their relative density, facilitates pore formation^25^. In sharp contrast, when syt1 was co-reconstituted into ND3_L_, robust pore formation occurred upon addition of Ca^2+^ (Fig. 1c); however, these pores exhibited rapid, dramatic flickering behavior.

We then assessed ND9_L_ (Fig. 1a; 0.013 syb2/nm^2^) lacking syt1; these NDs yielded pores that remained mostly in the open state but transiently flickered closed (Fig. 1d). In contrast, ND3_S_, which has a somewhat higher syb2 density, gave rise to pores that were mostly in closed state but transiently flickered open. We conclude that the higher syb2 copy number in ND9_L_ serves to hold the pore in a stable open state^16^. Strikingly, inclusion of syt1 in ND9_L_ potently inhibited pore formation in the absence of Ca^2+^ (Fig. 1d). These conditions appear to recapitulate the clamping activity of syt1 that has been observed in neurons^26–29^. Subsequent addition of Ca^2+^ triggered the opening of relatively stable, large pores (Fig. 1d).

To conduct quantitative analysis, we carried out 20–40 trials under each of the conditions described above. We first calculated the frequency of pore formation; these findings are represented as “percentage of occurrence” (Fig. 1e). This analysis demonstrated that the occurrence of pores formed by SNAREs alone was unaffected by Ca^2+^ (Fig. 1e). Moreover, inclusion of syt1 in both ND3_S_ and ND9_L_ reduced the occurrence of pores in the absence of Ca^2+^ (Fig. 1e). Interestingly, this clamping activity was the most apparent using ND9_L_, a condition that most closely mimics the syb2 density on SVs (0.014 syb2/nm^2^)^30^. Using ND3_L_, we could not address clamping activity because SNAREs alone failed to form pores; under this condition, inclusion of syt1 resulted in the appearance of small, unstable pores (Fig. 1e). In summary, the clamping activity of apo-syt1 is strongly dependent on both SNARE density and copy number (Supplementary Fig. 2). In contrast, Ca^2+^•syt1 clearly increased the occurrence of pores for all three types of NDs that were tested, as compared to the Ca^2+^-free BAPTA control condition.

**Fig. 2:**
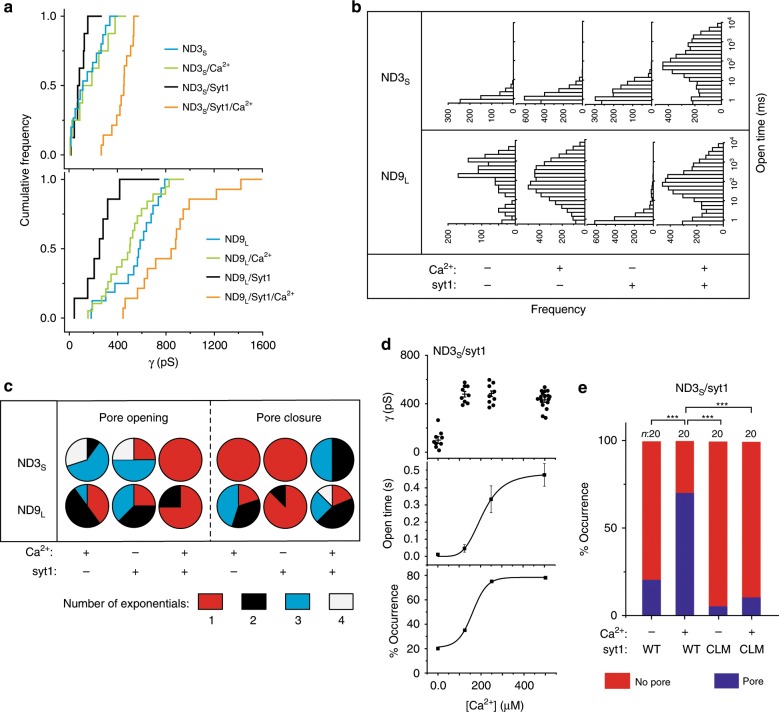
Quantification of syt1-regulated fusion pore properties. **a** Cumulative distribution functions (CDF) of single-channel conductances across different trials for each experimental condition. **b** Open dwell time histograms for ND3_S_ and ND9_L_, plus or minus syt1 and Ca^2+^, are shown. **c** Pie diagrams showing the number of exponentials required to fit the open- and closed-state CDFs for individual pores under the indicated conditions. Details are described in Supplementary Fig. 4. **d** Conductance values (*γ*) (upper panel), open time (middle panel), and percentage of occurrence (lower panel) at each indicated [Ca^2+^]_free_ are plotted. *n* *=* 16, 10, 10, 15; three different sets of ND preparations were used. In the open time plot, mean values for the open-state dwell times of individual traces were quantified. Error bars indicate SEM. **e** Percentage of occurrence of pore formation, plotted for WT syt1 versus a Ca^2+^ ligand mutant (CLM), D230/232N/D363/365N, that fails to bind Ca^2+^ via either C2 domain. The number of independent trials (*n*) are indicated in **e**. Pearson’s χ^2^ analysis was performed; ***p < 0.001.

We next determined whether syt1 affects pore size, as compared to pores formed by SNAREs alone (Fig. 2a); because pores did not form with ND3_L_ lacking syt1, we focused on ND3_S_ and ND9_L_. Pore diameter was estimated via conductance measurements, and these data, from all trials, are plotted in Fig. 2a. Because the length dimension of a fusion pore is not known, and because this parameter might change with tilting of the SNARE TMDs, the pore diameter values, based on conductance measurements, are only approximations. For ND3_S_ and ND9_L_ in the absence of syt1 and Ca^2+^, the mean conductance was 170 ± 32 and 563 ± 49 pS, respectively. Addition of Ca^2+^ had little effect (conductance values of 213 ± 56 and 501 ± 47 pS, respectively; Fig. 2a and Supplementary Fig. 3a). Inclusion of syt1 in ND3_S_ and ND9_L_ yielded mean pore conductance values of 105 ± 32 and 299 ± 74 pS, respectively, in the absence of Ca^2+^ (Fig. 2a and Supplementary Fig. 3a). Thus apo-syt1 unexpectedly decreases the size of fusion pores (see Supplementary Fig. 3b for estimates of pore size determined for all conditions described in this section; a graphical representation of these data is provided in Supplementary Fig. 13). In contrast, Ca^2+^ greatly increased the size of pores formed by syt1-bearing ND3_S_ and ND9_L_, yielding mean conductance values of 415 ± 19 and 902 ± 103 pS, respectively (Fig. 2a and Supplementary Fig. 3a). These results reveal that Ca^2+^•syt1 not only opens pores but also drives dilation (Fig. 2a) at both of the SNARE densities that were tested.

We then compared the kinetic properties of fusion pores formed under various conditions. Open time distribution plots demonstrated that Ca^2+^ had no effect on individual ND3_S_ pores (Fig. 2b, upper panel); inclusion of syt1 slightly shifted the distribution to shorter life times in BAPTA while addition of Ca^2+^ drastically increased the open life time (Fig. 2b, upper panel). In the case of ND9_L_ pores, the findings were more complicated (Fig. 2b, lower panel). Here Ca^2+^ had a small effect on individual ND9_L_ pores lacking syt1, and inclusion of syt1 shifted the distribution to shorter life times in BAPTA. However, as in the case with ND3_S_/syt1, addition of Ca^2+^ markedly increased the open life time of ND9_L_/syt1 pores (Fig. 2b, lower panel). Together, these results demonstrate that syt1 has dramatic, direct effects on the kinetic properties of individual, recombinant, fusion pores. Ca^2+^•syt1 not only increases the size of pores but also promotes membrane fusion by significantly stabilizing the open state.

In order to gain insights into the number of kinetic steps during pore transitions, the cumulative distribution functions (CDFs) for the opening and closure of individual pores were fitted with single or multiple exponential functions (Supplementary Figs. 4–6). CDFs of the closed and open time distributions reflect the kinetics of pore opening and closure, respectively. In the absence of syt1, pore opening was described by multiple exponentials for both the ND3_S_ and ND9_L_ (Fig. 2c); inclusion of apo-syt1 had little effect (Fig. 2c). In sharp contrast, Ca^2+^•syt1 resulted in mainly single exponential kinetics for pore opening, despite the SNARE copy number heterogeneity in our ND preparations (Fig. 2c and Supplementary Figs. 4–6). These findings indicate that Ca^2+^•syt1 drives fusion pores into a single open state with little or no involvement of intermediates.

Regarding pore closure, in the absence of syt1, single exponential kinetics were observed for ND3_S_, while ND9_L_ pores followed multi-exponential kinetics. In contrast, inclusion of apo-syt1 resulted in predominantly single exponential kinetics for both ND3_S_ and ND9_L_, even though the number of SNAREs remained the same. Finally, in the presence of syt1 and Ca^2+^, pore closure for ND3_S_ and ND9_L_ was best described by multiple exponentials (Fig. 2c and Supplementary Figs. 4–6). These findings indicate that pore closure, in the presence of Ca^2+^$\scriptstyle{\bullet}$syt1, occurs via a complex multi-state mechanism.

### Ca^2+^ dependencies of fusion pore parameters

We conducted [Ca^2+^] dose–response experiments to determine whether the effects of Ca^2+^ on fusion pore conductance, opening, and dilation described in Fig. 2 could be dissociated from one another. Representative traces of individual ND3_S_/syt1 pores as a function of [Ca^2+^]_free_ are shown in Supplementary Fig. 7a. The Ca^2+^-dependent effect of syt1 on pore conductance was saturated at 125 µM [Ca^2+^]_free_ (Fig. 2d, top panel); we were unable to reliably determine conductance values at lower [Ca^2+^], so these data were not fitted to estimate an EC_50_ or Hill slope. The open life time, however, continued to increase at higher [Ca^2+^], with an EC_50_ of 210 ± 60 µM and a Hill coefficient of 4.3 ± 0.4 (Fig. 2d, middle panel). Finally, we determined the fraction of trials in which a pore was observed and found that the EC_50_ was 164 ± 20 µM Ca^2+^ and the Hill coefficient was 3.4 ± 0.2 (Fig. 2d, lower panel). The apparent cooperativity in our experiments agree with the Hill slope for release as determined using neurons^31^. We note that all Ca^2+^ titration measurements were carried out using the same Ca^2+^ stocks and buffers. The different Ca^2+^ requirements for each of the fusion pore parameters suggest that different Ca^2+^-binding sites in syt1 (which binds 4-5 Ca^2+^ ions)^9,32,33^ might subserve distinct functions during exocytosis.

As a control, we tested a mutant form of syt1 in which two acidic Ca^2+^ ligands, in each C2-domain of syt1, were neutralized by substitution with asparagine residues, thus abolishing Ca^2+^-binding activity^34,35^. This Ca^2+^ ligand mutant was unable to couple Ca^2+^ to fusion pore opening (Fig. 2e).

### Syt1•PIP_2_ interactions stabilize fusion pores

**Fig. 3:**
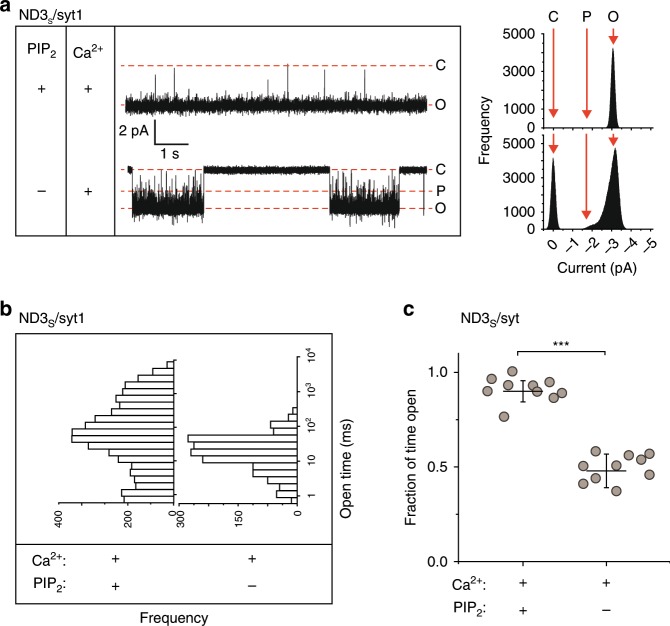
Syt1•PIP_2_ interactions stabilize the open state of fusion pores. **a** Traces of single pores, formed using ND3_S_/syt1 plus Ca^2+^, with (+, upper panel) and without (−, lower panel) 2% PIP_2_ in the BLM. Respective current histograms are shown beside the traces. Closed (C), open (O), and partially open (P) states are shown; the current and time scale applies to both traces. **b** Open dwell time histogram of pores (combined data from all trials), under the conditions described in **a**. **c** Fraction of time pores were open over a 30-min period, calculated from the data collected under the indicated conditions. Error bars indicate SEM from ten independent BLMs and three independent sets of each kind of ND; the Student’s *T* test was performed to compare the two means; ***p < 0.001.

It is established that syt1 must penetrate membranes that harbor acidic phospholipids in order to drive membrane fusion in vitro^36^ and to trigger exocytosis in cells^26,28,37^. There is evidence that PIP_2_, which is localized to the plasma membrane, interacts with syt1 under resting conditions to steer the Ca^2+^-triggered membrane penetration of its C2-domains toward the target membrane^38^ to regulate fusion in vitro^36^. Indeed, PIP_2_ plays an essential role in exocytosis in neuroendocrine cells^39,40^ and appears to play a key role in at least some modes of SV release^41^. However, before the development of the fully defined ND-BLM system, it had not been possible to directly explore the role of PIP_2_ on the biophysical properties of individual fusion pores, as previous work relied on cell-based measurements. We therefore carried out ND-BLM measurements using BLMs with and without PIP_2_. These experiments, using ND3_S_/Ca^2+^•syt1, revealed that, in the absence of PIP_2_, pores became less stable (Fig. 3a–c), with the appearance of a partially open state (Fig. 3a). Similar results were obtained using ND3_L_/Ca^2+^•syt1 but with a more prominent partially open state (Supplementary Fig. 7b, c). As a control, PIP_2_ had no effect on pores formed by ND3_S_ in the absence of syt1 (Supplementary Fig. 7d, e). Interesting, PIP_2_ had a minimal effect on pore occurrence (Supplementary Fig. 7f). As a further control, both PS and PIP_2_ were omitted from the BLM; under these conditions, Ca^2+^ and syt1 were unable to regulate pores (Supplementary Fig. 8). These experiments confirm that syt1–lipid interactions play a key role in the regulation of fusion pores.

### Ca^2+^•syt1 commits *trans*-SNARE complexes

We next turned to the question of how syt1 affects *trans*-SNARE complex assembly^42^, using a previously described smFRET approach^19^. It was not technically feasible to conduct smFRET using v-SNARE NDs bound to t-SNARE BLMs, so we used v- and t-SNARE NDs. The strength of this approach is that the assembly state of full-length, membrane-embedded SNAREs can be assessed, but we note that the t-SNARE NDs are more constrained as compared to the BLM. Nonetheless, this system revealed that apo- and Ca^2+^-bound syt1 have distinct, direct effects on the assembly of *trans*-SNARE complexes.

**Fig. 4:**
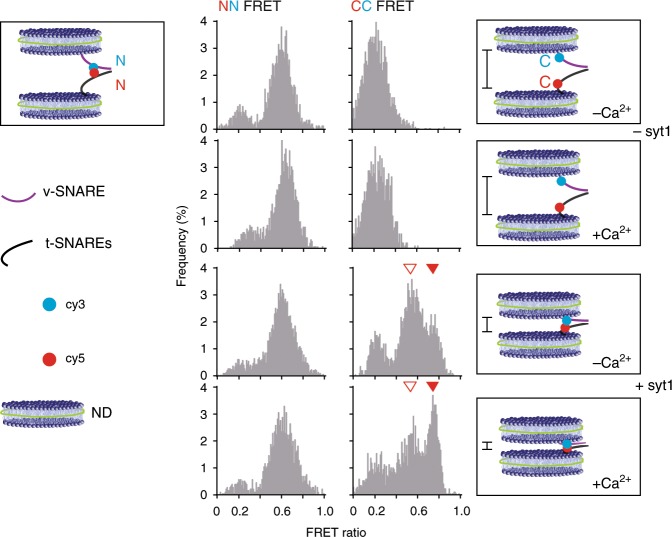
smFRET reveals the impact of syt1 on *trans*-SNARE complex assembly. smFRET histograms of *trans*-SNARE complexes, under the conditions indicated on the far right, using FRET pairs (cy3, shown as blue dots and cy5, shown as red dots) near the N- (NN; left panels) or the C-termini (CC; right panels) of the SNARE motifs of syb2 and syntaxin-1A, respectively. For clarity, the t-SNARE heterodimer is presented by a single black line, and because NN smFRET was not affected by Ca^2+^ or syt1, only the starting condition is illustrated for the NN experiments (upper left). In the illustrations on the right (+/− Ca^2+^ and syt1), vertical lines indicate the relative distance between v- and t-SNARE NDs under each condition. NN: *n* = 67 (−Ca^2+^, −syt1), 61 (+Ca^2+^, −syt1), 83 (−Ca^2+^, +syt1), 64 (+Ca^2+^, +syt1). CC: *n* = 79 (−Ca^2+^, −syt1), 69 (+Ca^2+^, −syt1), 74 (−Ca^2+^, +syt1), 87 (+Ca^2+^, +syt1). ND1_S_ were used in all experiments. Data were collected using three independent sets of NDs. In all smFRET experiments, −Ca^2+^ samples contained 1 mM EGTA; +Ca^2+^ samples contained 500 µM [Ca^2+^]_free_.

Syb2 was labeled with a donor fluorophore (cy3), and syntaxin-1A was labeled with an acceptor (cy5). Monitoring multiple *trans*-SNARE complexes by smFRET is extremely challenging, so we used ND1 for these experiments. For each SNARE, two positions were selected, based on the crystal structure of the *cis*-complex^43,44^: one pair at layer −7 near the N-termini of the SNARE motifs (N-N FRET pair), and the other between layer +4 and +5 near the C-termini of the SNARE motifs (C-C FRET; Supplementary Fig. 9a; Fig. 4). Labeled *trans*-SNARE complexes were first formed using v- and t-SNARE NDs in the absence of syt1. Under these conditions, the FRET ratio (*R*) distribution of the N-terminal pair peaked at ~0.7, while this value was only ~0.2 for the C-C pair; Ca^2+^ had no effect (Fig. 4). Thus single *trans*-SNARE complexes formed between two NDs in the absence of syt1 but exist in a partially zippered state: the N-termini of the SNARE motifs assembled together, but the C-termini remained unzipped. These findings indicate that one SNARE pair is not sufficient to drive fusion in this system, a finding that is also consistent with the inability of ND3_L_ to form viable pores (Fig. 1e). In the presence of apo-syt1 (reconstituted in the v-ND), the N-N pair remained in the high FRET state, whereas the C-C pair resulted in three different FRET states: 0.2, 0.5, and 0.7 (Fig. 4). These results demonstrate that apo-syt1 drives further assembly of the SNARE complex. We suggest that this structure corresponds to the primed, clamped conformation of the *trans*-SNARE complex. Subsequent addition of Ca^2+^ to these samples had only subtle effects on the smFRET signals (Fig. 4), probably because the C-terminal end of the SNARE motif had largely assembled into a primed and clamped structure^29,45^. However, an increase in the highest C-C FRET peak was apparent, revealing that Ca^2+^-syt1 drives further assembly of the SNARE complex toward the membrane anchors. For completeness, representative raw time-based FRET traces, showing all three FRET states, are shown in Supplementary Fig. 9b. We note that our smFRET experiments are in agreement with previous studies, based on force measurements, that examined the assembly of *trans*-SNARE complexes^46–48^ (Supplementary Fig. 9a). In short, the ability of syt1 to clamp membrane fusion prior to the Ca^2+^ signal is associated with its ability to drive assembly of *trans*-SNARE complexes into a more zippered, yet inhibited, state. Then addition of Ca^2+^ drives further assembly and thus fusion pore opening. These findings are consistent with cell-based studies which indicate that the C2B-domain of apo-syt1 is a potent fusion clamp^26,49^. This clamping activity appears to be controlled by conformational changes that determine the relative disposition of the tandem C2-domains. Namely, apo-syt1 clamps fusion when the tandem C2-domains of apo-syt1 are askew^26^. Then, upon binding Ca^2+^, the C2-domains reorient and point in the same direction to trigger exocytosis^26^.

**Fig. 5:**
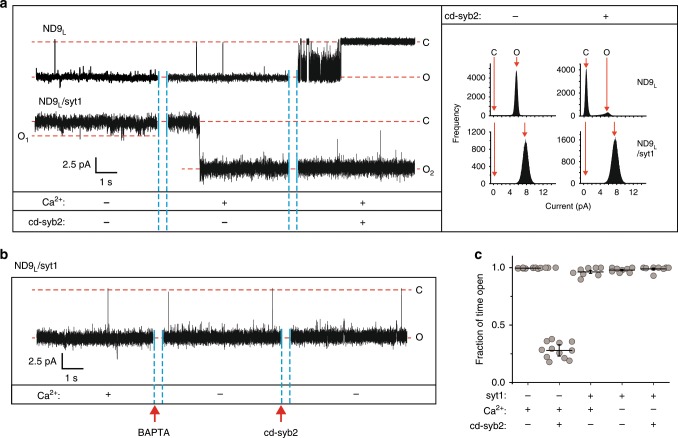
Ca^2+^•syt1 drives *trans*-SNARE complexes into a functionally committed state. **a** Representative recording of ND9_L_ lacking or containing syt1. Once pores were open, 20 µM cd-syb2 was added to each reaction. Closed (C) and open (O) states are indicated; the current/time scale for all traces is shown in the inset, left. Right panel: respective current histograms are shown. **b** Representative recordings of ND9_L_ bearing syt1 in 500 µM [Ca^2+^]_free_ are shown, followed by the addition of 1.5 mM BAPTA (arrow), further followed by 20 µM cd-syb2 (arrow). The current/time scale is shown on the bottom left. **c** Fraction of time individual pores were open over a 45-min period, plotted for each condition as indicated. Error bars indicate SEM from 12 and 8 independent BLMs for **a** and **b**, respectively; 3 independent sets of NDs were used.

In a second approach, we utilized the cytosolic domain of syb2 (cd-syb2), which competes with full-length syb2 in NDs for binding t-SNAREs in the BLM, to prevent or disrupt *trans*-SNARE pairing. In an earlier study, we found that, in the absence of syt1, cd-syb2 readily closes fusion pores, thus revealing that *trans*-SNARE complexes are not fully assembled, or committed, at a stage in which fusion pores have opened to a relatively small conductance^16^. Here we started with stable, open ND9_L_ pores lacking syt1 and, consistent with our previous study, observed that 20 µM cd-syb2 efficiently closed individual pores (Fig. 5a, upper panel); Ca^2+^ was without effect. We then carried out the same experiments using ND9_L_/syt1. After the pore was opened by Ca^2+^ and robust currents, indicative of large pores, were detected, 20 µM cd-syb2 was added. Remarkably, pore closure was not observed, even over a 45-min recording period (Fig. 5a, lower panel). We then repeated this experiment but first added excess BAPTA to chelate Ca^2+^ and deactivate syt1; even then, subsequent addition of cd-syb2 was without effect (Fig. 5b and Supplementary Fig. 10a, b). Thus the action of Ca^2+^•syt1 appears to be terminal, as the ongoing presence of Ca^2+^ is not required to maintain pore properties.

To formalize these observations, we calculated the fraction of time that individual pores were in the open state over a 45-min period. This fraction decreases in the presence of cd-syb2 when SNAREs alone were used (Fig. 5c). In contrast, after the action of Ca^2+^•syt1, the fraction open was unaffected by cd-syb2 (Fig. 5c). These results further demonstrate that syt1 drives assembly of *trans*-SNARE complexes such that they enter into a committed, in effect “irreversible,” state that requires additional energy to become disassembled.

### α-SNAP/NSF-ATP closes fusion pores

**Fig. 6:**
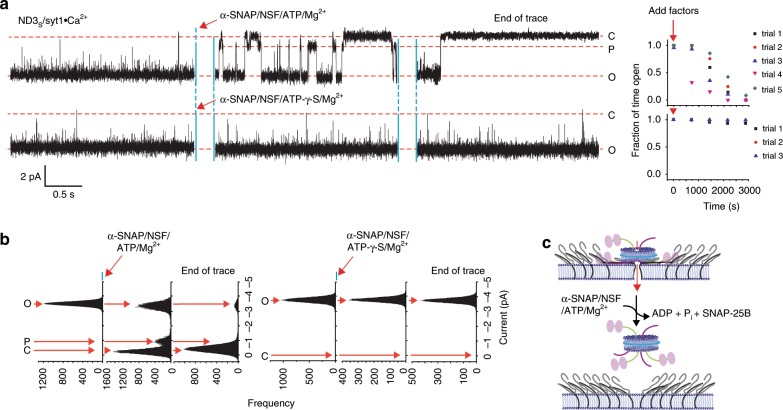
α-SNAP/NSF-ATP disassemble *trans*-SNARE complexes formed by Ca^2+^•syt1. **a** Representative recording of a pore formed by ND3_S_/syt1 in 500 µM [Ca^2+^]_free_. After pore formation, 0.3 µM α-SNAP, 0.3 µM NSF, 1 mM ATP, and 5 mM MgCl_2_ were added (red arrow). In the lower panel, ATP was replaced with 1 mM ATP-γ-S. Two different epochs of each trace are shown; the time interval between each epoch is indicated. Closed (C), open (O), and partially open (P) states are marked; the current/time scale for all traces is provided in the inset. After addition of the disassembly factors, recordings were divided arbitrarily into five 720-s epochs; the fraction of time the pores were open during each epoch was quantified for individual traces and plotted beside each panel. *n* = 5 BLMs using ATP, and *n* = 3 BLMs using ATP-γ-S; two different sets of NDs were used for each condition. **b** Representative current histograms for the experiments described above are shown. **c** Illustration (drawn to scale) shows the α-SNAP/NSF/ATP/Mg^2+^-mediated disassembly of SNAREs, leading to fusion pore closure.

Since fusion pores formed in the presence of Ca^2+^ and syt1 comprise committed *trans*-SNARE complexes, we examined whether they could be closed by NSF, an AAA+ ATPase that acts to disassemble SNARE complexes in vitro^11^ and in vivo^50^. NSF is recruited to SNARE complexes via an adaptor protein, α-SNAP^11,12^. Then, upon ATP hydrolysis by NSF, SNARE complexes are disassembled^11,51^. In vivo, NSF is thought to act on *cis*-SNARE complexes in the plasma membrane following fusion, where disassembly allows v- and t-SNAREs to be segregated into separate compartments for subsequent rounds of fusion. Whether NSF can act on *trans*-SNARE complexes has been the subject of debate^13,14^. The ND-BLM assay makes it possible to determine directly whether NSF acts on *trans*-SNARE complexes in a functional manner, as disassembly would be evidenced by pore closure. As shown in Fig. 6, addition of α-SNAP/NSF-ATP to fusion pores formed by ND3_S_/syt1•Ca^2+^ resulted in dramatic changes: rather than brief flickers to the closed state, prolonged closures became apparent, eventually leading to complete, irreversible closure. We note that a distinct subconductance state appeared in 67% of open-to-closed and 75% of closed-to-open transitions during NSF-mediated pore dissolution (Fig. 6a, b and Supplementary Fig. 11a). In all five trials, we observed repeated transitions between open/partially open and closed states before the final, irreversible, closure. We term this a “stuttering” mechanism for pore dissolution. Apparently, there are intermediate steps that are reversible during disassembly. Irreversible closure likely results from loss of SNAP-25B, which becomes too dilute after disassembly to efficiently re-bind syntaxin-1A. Use of the ATP analog ATP-γ-S (Fig. 6a, lower panel, 6a) or omission of Mg^2+^ (Supplementary Fig. 11b) abolished the action of α-SNAP/NSF-ATP on pore closure, confirming that disassembly depended on efficient hydrolysis of ATP. Finally, the individual components: α-SNAP, NSF-ATP, or MgCl_2_, had no appreciable effect on pores, demonstrating that all disassembly factors must be present at the same time in order to close pores (Supplementary Fig. 11c). Together, these results demonstrate that α-SNAP/NSF can close fusion pores, presumably by disassembly of committed *trans*-SNARE complexes, and this effect requires ATP hydrolysis by NSF (Fig. 6c). In summary, syt1 acts to promote SNARE complex assembly to the point where it requires additional energy, provided by the ATPase NSF, to disassemble.

## Discussion

At present, little is known concerning the structure and dynamics of *trans*-SNARE complexes because they have been difficult to trap in distinct functional states. Most of what is known regarding SNARE complex assembly and disassembly stems from studies based on *cis* complexes^43,44,52^, which do not form until after fusion. The major goal of the current study was to address the relationship between *trans*-SNARE complex assembly and fusion pore properties. We approached this question by determining how transitions in these structures are affected by two classes of regulatory proteins: one—syt1—is thought to trigger pore opening, while the other—α-SNAP/NSF—serves to disassemble SNARE complexes. To study kinetic transitions in pores, we applied a newly described ND-BLM method, which affords microsecond time resolution. This approach was combined with smFRET measurements to monitor SNARE zippering.

The first goal was to address the impact of syt1 on recombinant fusion pores. Under resting conditions, it has been proposed that apo-syt1 serves as a fusion clamp that prevents SV exocytosis until the arrival of an action potential and a concomitant increase in [Ca^2+^]_i_^26,28,29,49^. We observed that a clamping function for syt1 emerged in the ND-BLM system and was most prominent when fusion pores were formed using physiological densities of syb2^30^ (Figs. 1 and 2). smFRET measurements indicate that the ability of syt1 to clamp fusion, under resting conditions, was mediated by a direct action on *trans*-SNARE complexes (Fig. 4). In the absence of Ca^2+^, spontaneous openings still occurred to some degree, and kinetic analysis revealed that these openings followed multi-exponential kinetics (Fig. 2c), indicating the involvement of multiple intermediates. In contrast, in the presence of both Ca^2+^ and syt1, pore opening followed single exponential kinetics, suggesting that all *trans*-SNARE complexes were driven into the same primed state, making the pore opening the result of a single collective stroke. It will be interesting to determine which of the distinct syt1-SNARE complex structures that have been reported^45,53,54^ coincide with the primed but clamped state, and with the fusogenic state, of this protein complex.

Syt1 is unlikely to clamp fusion by preventing the docking of NDs to the BLM via steric effects, as this protein has been reported to facilitate docking in reconstituted systems and in synapses^36,55^. Moreover, we used physiologically relevant syb2 and syt1 densities that approximate the densities found on native SVs^30^. Indeed, SVs contain a myriad of additional proteins, so are more crowded than our NDs. Finally, the observation that syt1 inhibits pore activity, at least in part, by reducing the open life time in a dose-dependent manner (Supplementary Fig. 2) indicates that aspects of the inhibitory/clamping activity of syt1 are mediated by the intrinsic properties of the protein.

Upon addition of Ca^2+^, syt1 efficiently triggered fusion pore opening in the ND-BLM system, under all conditions that were tested. So we conducted experiments to probe for changes in the structure of the underlying SNARE complexes. We previously observed, using SNAREs alone, that addition of cd-syb2 resulted in the disassembly of *trans*-SNARE complexes and pore closure^16^. These findings prompt the question of how far pores must progress such that SNAREs assemble into highly stable state that requires the action of NSF in order to be disassembled. Indeed, Ca^2+^•syt1 not only opened pores but also drove pore dilation (Supplementary Fig. 12), and this was associated with further assembly of *trans*-SNARE pairs as revealed by smFRET. Remarkably, once pores were opened by Ca^2+^•syt1, they became resistant not only to a Ca^2+^ chelator but also to high concentrations of cd-syb2. Apparently, *trans*-SNARE complexes had entered into a committed state such that cd-syb2 could no longer displace membrane-anchored syb2, within SNARE complexes, even over relatively long time frames (45 min). Committed fusion pores still flickered, and this might reflect their partially lipidic structure^7,56,57^. We also note that the interaction of syt1 with PIP_2_^36,38^, a plasma membrane lipid that plays a key role in exocytosis, served to stabilize the Ca^2+^-triggered opening of fusion pores, so lipids also impact—directly or indirectly—fusion pore transitions.

Given that fusion pores triggered to open by the action of Ca^2+^•syt1 were functionally committed, we asked whether α-SNAP/NSF/ATP was able to disassemble the SNARE complexes that formed them. While it is well established that the action of α-SNAP and NSF serves to disassemble *cis*-SNARE complexes after fusion, whether these factors can disassemble *trans*-complexes remains unresolved^13,14^. The ND-BLM system provides a functional read-out that relates SNARE complex disassembly with fusion pore transitions in real time. Indeed, upon hydrolysis of ATP, NSF and α-SNAP did in fact close fusion pores formed by *trans*-SNARE complexes. This experiment further demonstrates that SNARE zippering and unzippering reactions underlie structural and kinetic transitions in individual fusion pores. Moreover, these findings support the conclusion that we are studying *trans*, and not *cis*, complexes, as disassembly of *cis* complexes would not be expected to close pores. It is possible that the TMD of syb2 binds to the MSP, preventing zippering with the TMD of syntaxin^43^, but we did not observe binding in pull-down assays (Supplementary Fig. 13). However, since these experiments were performed in the presence of detergent, it remains formally possible that interactions might occur within lipid-filled NDs. It is also possible that the curvature of the membranes, or the proteinaceous aspects of the pore, prevent the v- and t-SNARE TMDs from coalescing.

In all trials, NSF-mediated closure involved flickering behavior and the appearance of a subconductance state. This state was most commonly observed during pore transitions (Fig. 6a and Supplementary Fig. 11a). Because the partially open state was associated with both open-to-closed, as well as closed-to-open, transitions, it is not simply a product of vectorial disassembly. In this light, we note that cd-syb2 can give rise to subconductance states when used to close pores formed by SNAREs alone (Supplementary in ref. ^16^); this occurs via the formation of partially assembled SNARE complex intermediates. It is likely that α-SNAP/NSF acts in a similar manner, by allowing the forward formation of partially assembled *trans*-SNARE complexes, during ongoing disassembly, to yield a subconductance state. According to this view, there is a tug-of-war in which disassembly and assembly oppose one another, even during the ongoing action of NSF. One model to explain this tug-of-war is that the fusion pore is formed by two SNARE complexes. The action of NSF on one SNARE pair would close the pore, but the other SNARE complex would hold the ND in place so that the partially or completely disassembled pair can reassemble and thus re-open the pore. In this model, complete closure would occur when both SNARE pairs are disassembled at the same time. Regardless of the detailed underlying model, these time-resolved measurements indicate that functional disassembly of a pore is a complex process. Moreover, our findings raise the possibility that this disassembly reaction might underlie aspects of kiss-and-run exocytosis^8,15^. Interestingly, binding of SM (Sec1-Munc18) proteins in synapses^58^ or the binding of HOPS (Homotypic fusion and protein sorting) complex in case of vacuolar fusion^59^ might protect *trans-SNARE* complexes from NSF/α-SNAP-mediated disassembly^60^. Future studies will reveal whether these factors affect NSF-mediated fusion pore closure in the ND-BLM system described here.

In summary, we report key kinetic intermediates that are formed during the regulated assembly and disassembly of fusion pores. During assembly, the major Ca^2+^ sensor for SV exocytosis, syt1, directly regulates the occurrence, size, and dynamics of fusion pores by promoting SNARE zippering into a functionally committed state. This committed state could only be disassembled upon ATP hydrolysis by NSF, resulting in pore closure. Thus the defined, reduced, reconstituted ND-BLM system makes it possible to measure, in real time, the entire SNARE cycle, while revealing the behavior of fusion pores as they are constructed and deconstructed.

## Methods

### Materials

1-Palmitoyl-2-oleoyl-sn-glycero-3-phosphocholine (PC), 1,2-dioleoyl-sn-glycero-3-phospho-l-serine (PS), 1,2-dioleoyl-sn-glycero-3-phosphoethanolamine (PE), 1,2-diphytanoyl-sn-glycero-3-phosphocholine (DPhPC), 1-palmitoyl-2-oleoyl-sn-glycero-3-phospho-(1’-rac-glycerol) (sodium salt) (PG), and brain PI(4,5)P2 were purchased from Avanti polar lipids; DDM (n-dodecyl β-d-maltoside) and OG (n-octyl glucoside) were from Gold Biotechnology; IPTG was from Research Products International Corp.; Triton X-100 was from Sigma Aldrich; Ni-Sepharose 6 Fast Flow was from GE Healthcare; 2-mercaptoethanol and glycerol were from Thermo Fischer Sc.; Bio-beads SM2 were from BIO-RAD; ATP-γ-S was from Abcam.

### Protein purification

All cDNA used in this study was derived from rat. Syb2, t-SNARE heterodimers comprising syntaxin-1A and SNAP-25B, and full-length syt1 were expressed and purified as his_6_-tagged proteins, as described previously^7,36^. Syb2 and t-SNAREs were expressed at 37 °C in *Escherichia coli* BL21 (DE3) cells (ThermoFisher Scientific, Catalog number: C600003), whereas syt1 was expressed at 28 °C. In brief, bacterial pellets were resuspended (~10 ml per liter of culture) in resuspension buffer (25 mM HEPES-KOH [pH 7.4], 400 mM KCl, 10 mM imidazole, and 5 mM β-mercaptoethanol) and incubated for 20 min on ice after addition of 0.5 mg/ml lysozyme. Protease inhibitor (1 mM PMSF), DNase I, and RNase (Sigma, 10 µg/ml) were then added and samples were sonicated in 35 ml batches on ice for 2× 45 s (50% duty cycle). Triton X-100 was added to 2.1% (v/v) and incubated overnight with rotation at 4 °C before centrifugation of the cell lysate at 19,000 × *g* for 30 min in a JA-17 rotor (Beckman). The supernatant was then incubated for >2 h at 4 °C with Ni-NTA agarose (Qiagen; 0.5 ml of a 50% slurry per liter of cell culture) equilibrated in resuspension buffer. Beads were washed extensively with resuspension buffer containing 1% Triton X-100 and then washed with OG wash buffer (25 mM HEPES-KOH [pH 7.4], 400 mM KCl, 50 mM imidazole, 10% glycerol, 5 mM βmercaptoethanol, 1% octyl glucoside). The slurry was loaded onto a column, washed with 5–10 column volumes of OG wash buffer, and step-eluted with OG wash buffer containing 500 mM imidazole.

NSF, α-SNAP, the cytoplasmic domain of syb2, and membrane scaffold proteins (MSP1E3D1 and NW30) were also purified as his6-tagged proteins, as described previously^12,16,61,62^. In brief, a similar procedure as above was used to purify these proteins except all detergents were omitted from the wash buffers. The purified proteins were dialyzed against 25 mM HEPES-KOH (pH 7.4), 100 mM KCl, 10% glycerol, and 1 mM dithiothreitol (DTT).

### Proteoliposome reconstitution

t-SNARE liposomes were prepared as described previously. In brief, t-SNARE heterodimers were mixed together with lipids (25% PE, 75% PG) in reconstitution buffer (25 mM HEPES, pH 7.5, 100 mM KCl, and 1 mM DTT) plus 0.02% DDM. Detergent was removed with BioBeads under gentle shaking (overnight, 4 °C). t-SNARE liposomes were then isolated by flotation^16^, followed by dialysis against reconstitution buffer (overnight, 4 °C).

### ND reconstitution

Reconstitution of syb2 into NDs was performed as described^7,62^. In some experiments, full-length recombinant syt1^36^ was co-reconstituted with syb2 at a 1:1 molar ratio unless otherwise indicated. MSP1E3D1 was used to generate small, 13 nm, NDs (ND_S_) and NW30 was used to prepare large, 30 nm, NDs (ND_L_). The ratios of MSP to lipid molecules were 2:120 for ND_S_ and 2:1000 for ND_L_; the MSP-to-syb2 ratios were 2:1 (ND3) and 2:8 (ND9). The copy number of syb2 and syt1 per ND refers to the total number of syb2 and syt1 molecules, not the number of copies per face of the ND. The lipid composition was 40% PS, 45% PC, and 15% PE. Briefly, reconstitution involved mixing syb2, MSP, and lipids, with or without syt1 in reconstitution buffer containing 0.02% DDM. Detergent was slowly removed with BioBeads (1/3 volume, BIORAD) with gentle shaking (overnight, 4 °C). The preparation was centrifuged (20 min at 100,000 × *g*) to remove aggregates, and the NDs in the supernatant were purified by gel filtration using a Superdex 200 10/300 GL column, equilibrated in reconstitution buffer plus 5% glycerol.

### Planar lipid bilayer electrophysiology

Planar lipid bilayer recordings were performed using a Planar Lipid Bilayer Workstation from Warner Instruments (USA) as described^16,63,64^. Briefly, lipids (30% DOPE, 52% DPhPC, 16% DOPS and 2% brain PIP_2_, at 30 mg/ml in *n*-decane) were first painted onto a 150-μm aperture in a 1-ml, white Delrin or polystyrene cup (Warner Instruments) and dried for 15 min. Then the aperture was bathed in 1 ml of 25 mM HEPES, pH 7.4 with 100 mM KCl in the *cis* and 10 mM KCl in the *trans* chamber. The lipid solution was gently re-applied to the hole until a conductance-blocking seal was formed, as determined by capacitance measurements. This process was repeated, either with a brush or air bubble, until the desired capacitance was achieved.

### Single-channel measurements of fusion pores

After BLM formation, t-SNARE proteoliposomes (75% PE and 25% PG) were added to the *cis* chamber of the apparatus; these spontaneously fuse with the planar bilayer, thus depositing the t-SNAREs into the BLM. t-SNAREs were reconstituted into BLMs, at a density of 0.4 molecules per μm^2,16^. In our experiments, ~40 t-SNARE vesicles (of 40 to 50 nm diameter) fuse with the BLM^16^, altering the lipid composition of the BLM by only 0.0018%. Then, to form fusion pores, v-SNARE NDs (reconstituted with/without syt1) were added to the *cis* chamber. Pores form within 10–40 min and could be monitored for >90 min, before terminal closure. Currents were recorded using Bilayer Clamp Amplifier BC-535 (Warner Instrument) and a Digidata 1550B (with Humsilencer) acquisition system (Molecular Devices Corp.). Single-channel recordings were acquired at 10 kHz using the pCLAMP 10 (Molecular Devices, LLC.) software and were filtered at 5 kHz using a multisection Bessel filter. Δ*ψ* ≡ *ψ*_cis_ − *ψ*_trans_ (*ψ*_trans_ ≡ 0 V). All recordings were conducted at room temperature.

Experiments were initiated with 1 mM BAPTA in the *cis* chamber, followed by the sequential addition of reconstituted liposomes bearing t-SNAREs and then NDs. This was followed by the addition of 1.5 mM CaCl_2_ in the *cis*-chamber to yield 500 µM [Ca^2+^]_free_. To generate dose–response curves, different concentrations of CaCl_2_ were added to yield the indicated [Ca^2+^]_free_ in Fig. 2d and Supplementary Fig. 7a. Pore formation and dynamics were studied at Δ*ψ* = −60 mV.

To study the effect of PIP_2_ on fusion pores in Fig. 3 and Supplementary Fig. 7b–f, the lipid composition of t-SNARE proteoliposomes and NDs were kept constant, but the BLM lipid composition was varied as indicated in the text. BLMs were formed following same technique as described above.

To study the effect of cd-syb2, BAPTA, α-SNAP, NSF, ATP, and Mg^2+^, the indicated concentration of each factor was added to the *cis*-chamber, after pores had opened.

### Single-channel analysis of fusion pore unitary currents

All single-channel data were analyzed using Clampfit 10.7 (Molecular Devices, LLC.) and MS Origin 2016 (OriginLab, USA). In all figures showing BLM recordings, the representative traces were filtered at 1 kHz for display purposes.

Current histograms were plotted using CLAMPFIT 10.7 and fitted with Gaussian functions. To calculate the open life time of individual pores, 30-min periods (unless otherwise indicated) from individual traces were analyzed. Dwell times corresponding to the fully open and fully closed states were measured in individual records using the event detector in CLAMPFIT10.7. Open-state dwell time histograms were plotted using MS Origin 2016 (OriginLab, USA).

The fraction of time fully open was calculated using the equation:

1 $$\frac{{{\rm{Open}}\;{\rm{{dwell}}}\;{\rm{{time}}}}}{{{\rm{Open}}\;{\rm{dwell}}\;{\rm{time}} + {\rm{closed}}\;{\rm{dwell}}\;{\rm{time}}}}$$

### Kinetic analysis of single-channel data

Single-channel kinetic analysis was performed using previously reported methods^65^. Closed-state (*t*_closed_) and open-state (*t*_open_) dwell times detected in CLAMPFIT10.7 were statistically analyzed by CDFs [defined by the probability *P*(*t*_closed_ ≤ *t*) and *P*(*t*_open_ ≤ *t*)] using Origin 2016. Closed- and open-state CDFs of individual traces were fitted separately; representative CDFs with the different exponential fits are shown in Supplementary Fig. 4. CDFs generated from the closed- and open-state dwell times provided kinetics for the pore opening and pore closure, respectively. Exponential equations used to fit the CDFs are provided here.

2 $${\mathrm{One}}\;{\mathrm{exponential}}\;{\mathrm{function}}:{\mathrm{CDF}} = 1-{\mathrm{exp}}\left( { - kt} \right).$$

3 $${\mathrm{Two}}\;{\mathrm{exponential}}\;{\mathrm{function}}:{\mathrm{CDF}} = 1-{\mathrm{A}}_1{\mathrm{exp}}\left( { - k_1t} \right)-{\mathrm{A}}_2{\mathrm{exp}}\left( { - k_2t} \right).$$

4 $${\mathrm{Three}}\;{\mathrm{exponential}}\;{\mathrm{function}}:{\mathrm{CDF}} =	\, 1-{\mathrm{A}}_1{\mathrm{exp}}\left( { - k_1t} \right)-{\mathrm{A}}_2{\mathrm{exp}}\left( { - k_2t} \right)\\ 	-{\mathrm{A}}_3{\mathrm{exp}}\left( { - k_3t} \right).$$

5 $${\mathrm{Four}}\;{\mathrm{exponential}}\;{\mathrm{function}}:{\mathrm{CDF}} =	\, 1-{\mathrm{A}}_1{\mathrm{exp}}\left( { - k_1t} \right)-{\mathrm{A}}_2{\mathrm{exp}}\left( { - k_2t} \right)\\ 	-{\mathrm{A}}_3{\mathrm{exp}}\left( { - k_3t} \right)-{\mathrm{A}}_4{\mathrm{exp}}\left( { - k_4t} \right).$$

6 $${\mathrm{Five}}\;{\mathrm{exponential}}\;{\mathrm{function}}:{\mathrm{CDF}} =	\, 1-{\mathrm{A}}_1{\mathrm{exp}}\left( { - k_1t} \right)-{\mathrm{A}}_2{\mathrm{exp}}\left( { - k_2t} \right)-{\mathrm{A}}_3{\mathrm{exp}}\left( { - k_3t} \right)\\ 	-{\mathrm{A}}_4{\mathrm{exp}}\left( { - k_4t} \right)-{\mathrm{A}}_5{\mathrm{exp}}\left( { - k_5t} \right).$$

Akaike Information Criterion was used to determine the goodness of fit^65,66^;

7 $${\mathrm{AIC}} = n\;{\rm{log}}\left( {{\rm{RSS}}/n} \right) + 2\left( {k + 1} \right).$$

RSS in Eq. 7 is the residual sum of squares, *n* is the number of data points, and *k* is the number of parameters in each model tested.

### Single-molecule FRET measurements

Flow cells and 13 nm NDs bearing labeled SNAREs for single-molecule experiments were prepared as described previously^19,67^. Native cys residues were replaced with serines, and then cys residues were introduced for chemical labeling: syb2 was labeled at positions 33 or 72 with cy3 and syntaxin-1A was labeled at positions 203 or 241 with cy5. v- and t-SNARE NDs (5 µM) were incubated at room temperature for 30 min in the reconstitution buffer supplemented with 0.5 mM EGTA or Ca^2+^. This is similar to the waiting time for fusion pore formation in the ND-BLM system (10–30 min). Samples were then diluted to 10 pM before injection into flow cells. Unbound NDs were washed out and samples were imaged in imaging buffer consisting of (in mM): 1 Trolox, 0.5 CaCl_2_, 100 KCl, 25 HEPES, and an oxygen scavenging system (1% glucose, 1 mg/ml glucose oxidase, and 0.02 mg/ml catalase), pH 7.4. For experiments carried out in the absence of Ca^2+^, samples included 0.5 mM EGTA. Single-molecule imaging experiments were performed using a prism-based total internal reflection fluorescence microscope with 100-ms time resolution^19^. The FRET ratio (*R*) was calculated using the following equation:

8 $$R = \frac{{{\rm{IA}} - 0.05{\rm{ID}}}}{{{\rm{ID}} + {\rm{IA}}}}$$

In Eq. 8, ID and IA are the donor and acceptor intensities after background subtraction. Single-molecule spots were identified by alternating the 532 and 653 nm laser excitation to confirm the presence of both v- and t-SNARE NDs. Traces showing a single photobleaching event were selected for analysis. Each experiment was repeated at least three times independently, and one data set for each condition is presented. smFRET data were processed using custom MATLAB scripts. We fitted the FRET histograms with two- or three-component Gaussians to derive the FRET ratio (*R*) for each state.

### Statistical analysis

The number of independent trials is provided in the figure legends, along with the statistical tests that were performed. Error bars represent SEM.

## Supplementary information

Supplementary Information

Peer Review File

## Data Availability

Data supporting the findings of this manuscript are available from the corresponding author upon reasonable request. A reporting summary for this article is available as a Supplementary Information file. The source data underlying Figs. 1e, 2a, c, e, 3c, and 5c and Supplementary Figs. 3a, b, 7f, and 11a are provided as a Source Data file.

## Source data

Source data file

## References

[1] Ferro-Novick S, Jahn R (1994). Vesicle fusion from yeast to man. Nature.

[2] Sollner T (1993). SNAP receptors implicated in vesicle targeting and fusion. Nature.

[3] Weber T (1998). SNAREpins: minimal machinery for membrane fusion. Cell.

[4] McNew JA (2000). Compartmental specificity of cellular membrane fusion encoded in SNARE proteins. Nature.

[5] Breckenridge LJ, Almers W (1987). Currents through the fusion pore that forms during exocytosis of a secretory vesicle. Nature.

[6] Lindau M, Almers W (1995). Structure and function of fusion pores in exocytosis and ectoplasmic membrane fusion. Curr. Opin. Cell Biol..

[7] Bao H (2016). Exocytotic fusion pores are composed of both lipids and proteins. Nat. Struct. Mol. Biol..

[8] Chang C-W, Chiang C-W, Jackson MB (2017). Fusion pores and their control of neurotransmitter and hormone release. J. Gen. Physiol..

[9] Brose N, Petrenko AG, Sudhof TC, Jahn R (1992). Synaptotagmin: a calcium sensor on the synaptic vesicle surface. Science.

[10] Chapman ER (2008). How does synaptotagmin trigger neurotransmitter release?. Annu. Rev. Biochem..

[11] Sollner T, Bennett MK, Whiteheart SW, Scheller RH, Rothman JE (1993). A protein assembly-disassembly pathway in vitro that may correspond to sequential steps of synaptic vesicle docking, activation, and fusion. Cell.

[12] Zhao M (2015). Mechanistic insights into the recycling machine of the SNARE complex. Nature.

[13] Ungermann C, Sato K, Wickner W (1998). Defining the functions of trans-SNARE pairs. Nature.

[14] Weber T (2000). SNAREpins are functionally resistant to disruption by NSF and alphaSNAP. J. Cell Biol..

[15] Alabi AA, Tsien RW (2013). Perspectives on kiss-and-run: role in exocytosis, endocytosis, and neurotransmission. Ann. Rev. Physiol..

[16] Bao H (2018). Dynamics and number of trans-SNARE complexes determine nascent fusion pore properties. Nature.

[17] Wu Z (2016). Nanodisc-cell fusion: control of fusion pore nucleation and lifetimes by SNARE protein transmembrane domains. Sci. Rep..

[18] Wu, Z. et al. Dilation of fusion pores by crowding of SNARE proteins. *eLife* **6**, e22964 (2017). 10.7554/eLife.22964 PMC5404929 28346138

[19] Shin J, Lou X, Kweon D-H, Shin Y-K (2014). Multiple conformations of a single SNAREpin between two nanodisc membranes reveal diverse pre-fusion states. Biochem. J..

[20] Rao TC (2014). Distinct fusion properties of synaptotagmin-1 and synaptotagmin-7 bearing dense core granules. Mol. Biol. Cell.

[21] Rao TC (2017). Synaptotagmin isoforms confer distinct activation kinetics and dynamics to chromaffin cell granules. J. Gen. Physiol..

[22] Wang CT (2001). Synaptotagmin modulation of fusion pore kinetics in regulated exocytosis of dense-core vesicles. Science.

[23] Zhang Z, Hui E, Chapman ER, Jackson MB (2010). Regulation of exocytosis and fusion pores by synaptotagmin-effector interactions. Mol. Biol. Cell.

[24] Shi L (2012). SNARE proteins: one to fuse and three to keep the nascent fusion pore open. Science.

[25] Sharma S, Lindau M (2018). Molecular mechanism of fusion pore formation driven by the neuronal SNARE complex. Proc. Natl Acad. Sci. USA.

[26] Bai H (2016). Different states of synaptotagmin regulate evoked versus spontaneous release. Nat. Commun..

[27] Broadie K, Bellen HJ, DiAntonio A, Littleton JT, Schwarz TL (1994). Absence of synaptotagmin disrupts excitation-secretion coupling during synaptic transmission. Proc. Natl Acad. Sci. USA.

[28] Liu H (2014). Linker mutations reveal the complexity of synaptotagmin 1 action during synaptic transmission. Nat. Neurosci..

[29] Chicka MC, Hui E, Liu H, Chapman ER (2008). Synaptotagmin arrests the SNARE complex before triggering fast, efficient membrane fusion in response to Ca2. Nat. Struct. Mol. Biol..

[30] Takamori S (2006). Molecular anatomy of a trafficking organelle. Cell.

[31] Dodge FA, Rahamimoff R (1967). Co-operative action a calcium ions in transmitter release at the neuromuscular junction. J. Physiol..

[32] Fernandez I (2001). Three-dimensional structure of the synaptotagmin 1 C2B-domain: synaptotagmin 1 as a phospholipid binding machine. Neuron.

[33] Ubach J, Zhang X, Shao X, Sudhof TC, Rizo J (1998). Ca2+ binding to synaptotagmin: how many Ca2+ ions bind to the tip of a C2-domain?. EMBO J..

[34] Earles CA, Bai J, Wang P, Chapman ER (2001). The tandem C2 domains of synaptotagmin contain redundant Ca2+ binding sites that cooperate to engage t-SNAREs and trigger exocytosis. J. Cell Biol..

[35] Evans CS (2016). Functional analysis of the interface between the tandem C2 domains of synaptotagmin-1. Mol. Biol. Cell.

[36] Wang Z, Liu H, Gu Y, Chapman ER (2011). Reconstituted synaptotagmin I mediates vesicle docking, priming, and fusion. J. Cell Biol..

[37] Evans CS, Ruhl DA, Chapman ER (2015). An engineered metal sensor tunes the kinetics of synaptic transmission. J. Neurosci..

[38] Bai J, Tucker WC, Chapman ER (2004). PIP2 increases the speed of response of synaptotagmin and steers its membrane-penetration activity toward the plasma membrane. Nat. Struct. Mol. Biol..

[39] Eberhard DA, Cooper CL, Low MG, Holz RW (1990). Evidence that the inositol phospholipids are necessary for exocytosis. Loss of inositol phospholipids and inhibition of secretion in permeabilized cells caused by a bacterial phospholipase C and removal of ATP. Biochem. J..

[40] Hay JC (1995). ATP-dependent inositide phosphorylation required for Ca2+-activated secretion. Nature.

[41] Di Paolo G (2004). Impaired PtdIns(4,5)P2 synthesis in nerve terminals produces defects in synaptic vesicle trafficking. Nature.

[42] Bhalla A, Chicka MC, Tucker WC, Chapman ER (2006). Ca2+-synaptotagmin directly regulates t-SNARE function during reconstituted membrane fusion. Nat. Struct. Mol. Biol..

[43] Stein A, Weber G, Wahl MC, Jahn R (2009). Helical extension of the neuronal SNARE complex into the membrane. Nature.

[44] Sutton RB, Fasshauer D, Jahn R, Brunger AT (1998). Crystal structure of a SNARE complex involved in synaptic exocytosis at 2.4 A resolution. Nature.

[45] Zhou Q (2017). The primed SNARE-complexin-synaptotagmin complex for neuronal exocytosis. Nature.

[46] Gao Y (2012). Single reconstituted neuronal SNARE complexes zipper in three distinct stages. Science.

[47] Zorman S (2014). Common intermediates and kinetics, but different energetics, in the assembly of SNARE proteins. eLife.

[48] Ma, L. et al. Munc18-1-regulated stage-wise SNARE assembly underlying synaptic exocytosis. *eLife* **4**, e09580 (2015). 10.7554/eLife.09580 PMC4744192 26701912

[49] Courtney, N. A., Bao, H., Briguglio, J. S. & Chapman, E. R. Synaptotagmin 1 clamps synaptic vesicle fusion in mammalian neurons independent of complexin. *Nat. Commun.* **10**, 4076 (2019). 10.1038/s41467-019-12015-w PMC6733930 31501440

[50] Littleton JT (2001). SNARE-complex disassembly by NSF follows synaptic-vesicle fusion. Proc. Natl Acad. Sci. USA.

[51] Ryu J-K (2015). Spring-loaded unraveling of a single SNARE complex by NSF in one round of ATP turnover. Science.

[52] Fasshauer D, Antonin W, Subramaniam V, Jahn R (2002). SNARE assembly and disassembly exhibit a pronounced hysteresis. Nat. Struct. Mol. Biol..

[53] Brewer KD (2015). Dynamic binding mode of a Synaptotagmin-1-SNARE complex in solution. Nat. Struct. Mol. Biol..

[54] Zhou Q (2015). Architecture of the synaptotagmin-SNARE machinery for neuronal exocytosis. Nature.

[55] Reist NE (1998). Morphologically docked synaptic vesicles are reduced in synaptotagmin mutants of Drosophila. J. Neurosci..

[56] Chanturiya A, Chernomordik LV, Zimmerberg J (1997). Flickering fusion pores comparable with initial exocytotic pores occur in protein-free phospholipid bilayers. Proc. Natl Acad. Sci. USA.

[57] Fang Q (2008). The role of the C terminus of the SNARE protein SNAP-25 in fusion pore opening and a model for fusion pore mechanics. Proc. Natl Acad. Sci. USA.

[58] Ma C, Su L, Seven AB, Xu Y, Rizo J (2013). Reconstitution of the vital functions of Munc18 and Munc13 in neurotransmitter release. Science.

[59] Schwartz, M. L. et al. Sec17 (alpha-SNAP) and an SM-tethering complex regulate the outcome of SNARE zippering in vitro and in vivo. *eLife* **6**, e27396 (2017). 10.7554/eLife.27396 PMC5643095 28925353

[60] Carr CM, Rizo J (2010). At the junction of SNARE and SM protein function. Curr. Opin. Cell Biol..

[61] Nasr ML (2017). Covalently circularized nanodiscs for studying membrane proteins and viral entry. Nat. Methods.

[62] Shi L (2012). SNARE proteins: one to fuse and three to keep the nascent fusion pore open. Science.

[63] Finkelstein A (1974). Bilayers: formation, measurements, and incorporation of components. Methods Enzymol..

[64] Mueller P, Rudin DO, Tien HT, Wescott WC (1962). Reconstitution of cell membrane structure in vitro and its transformation into an excitable system. Nature.

[65] Das D, Krantz BA (2016). Peptide- and proton-driven allosteric clamps catalyze anthrax toxin translocation across membranes. Proc. Natl Acad. Sci. USA.

[66] Akaike, H. Citation classic - a new look at the statistical-model identification. *Curr. Contents Eng. Technol. Appl. Sci*. **51**, 22 (1981).

[67] Lou X, Shin J, Yang Y, Kim J, Shin Y-K (2015). Synaptotagmin-1 is an antagonist for Munc18-1 in SNARE zippering. J. Biol. Chem..

