# Supplementary material for: Resolving kinetic intermediates during the regulated assembly and disassembly of fusion pores
Source: Nat Commun. 2020 Jan 13;11:231. doi: 10.1038/s41467-019-14072-7 (PMC6957489; doi:10.1038/s41467-019-14072-7)
Supplement: Supplementary file 1 — Supplementary Information [file 41467_2019_14072_MOESM1_ESM.docx]

**Supplementary Information**


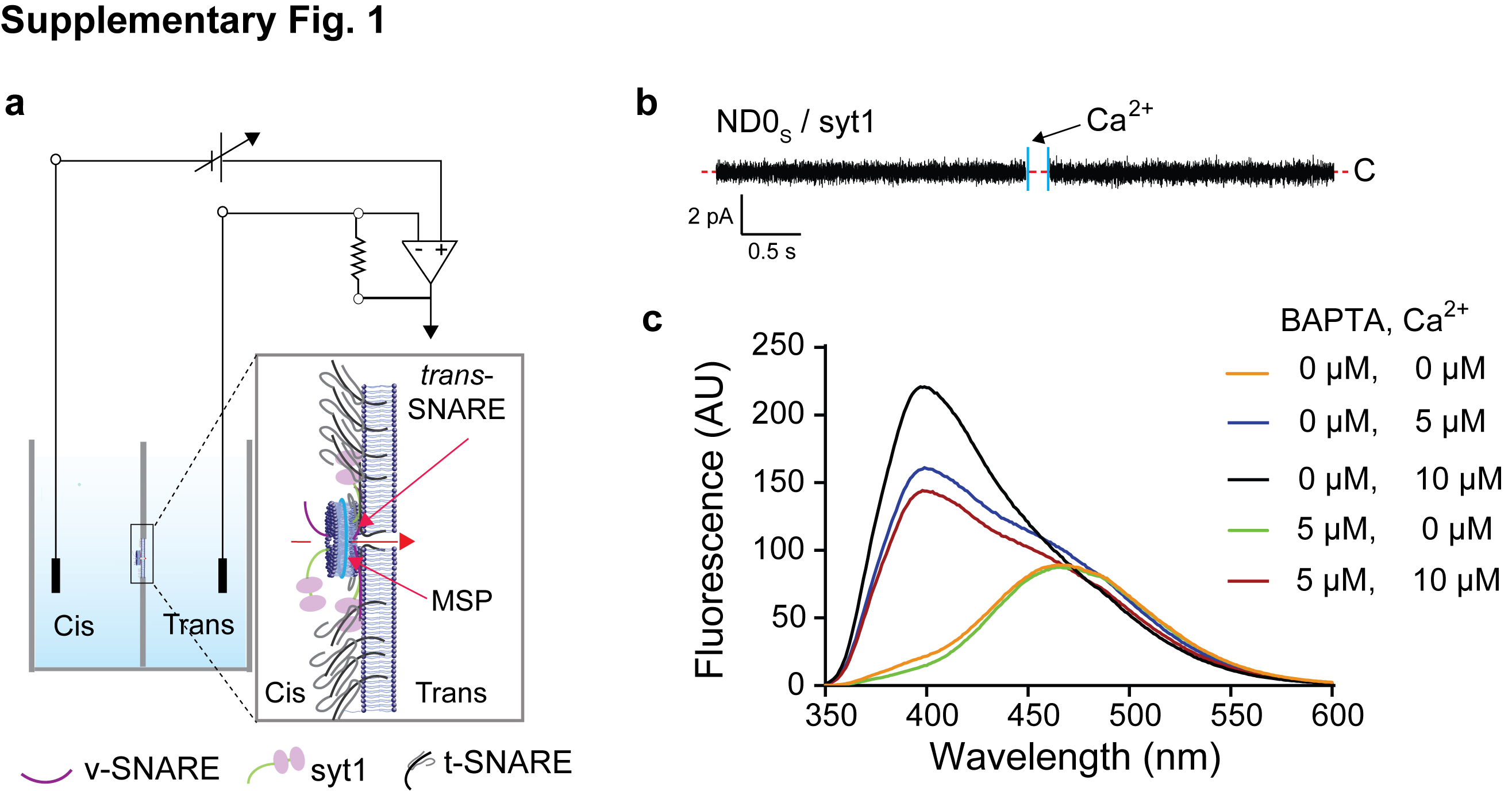


**Supplementary Fig.1. ND-BLM set-up, failure of syt1 NDs to form fusion pores in the absence of syb2, and determination of BAPTA-Ca^2+^ stoichiometry.**

**a,** Experimental setup for the ND-BLM system. **b,** Representative trace using small NDs lacking syb2 but bearing syt1 (ND0_S_/syt1), before and after addition of 500 μM [Ca^2+^]_free_ (indicated by an arrow); Δψ = -60 mV. Pores were not observed in 20 independent trials conducted using three different ND preparations that lacked syb2. (C), closed state; the current/time scale is shown in the inset. **c,** BAPTA-Ca^2+^ stoichiometry determination by Indo 1 (10 µM) fluorescence; excitation was at 340 nm. The [BAPTA] and [Ca^2+^] under each condition are indicated within the figure.


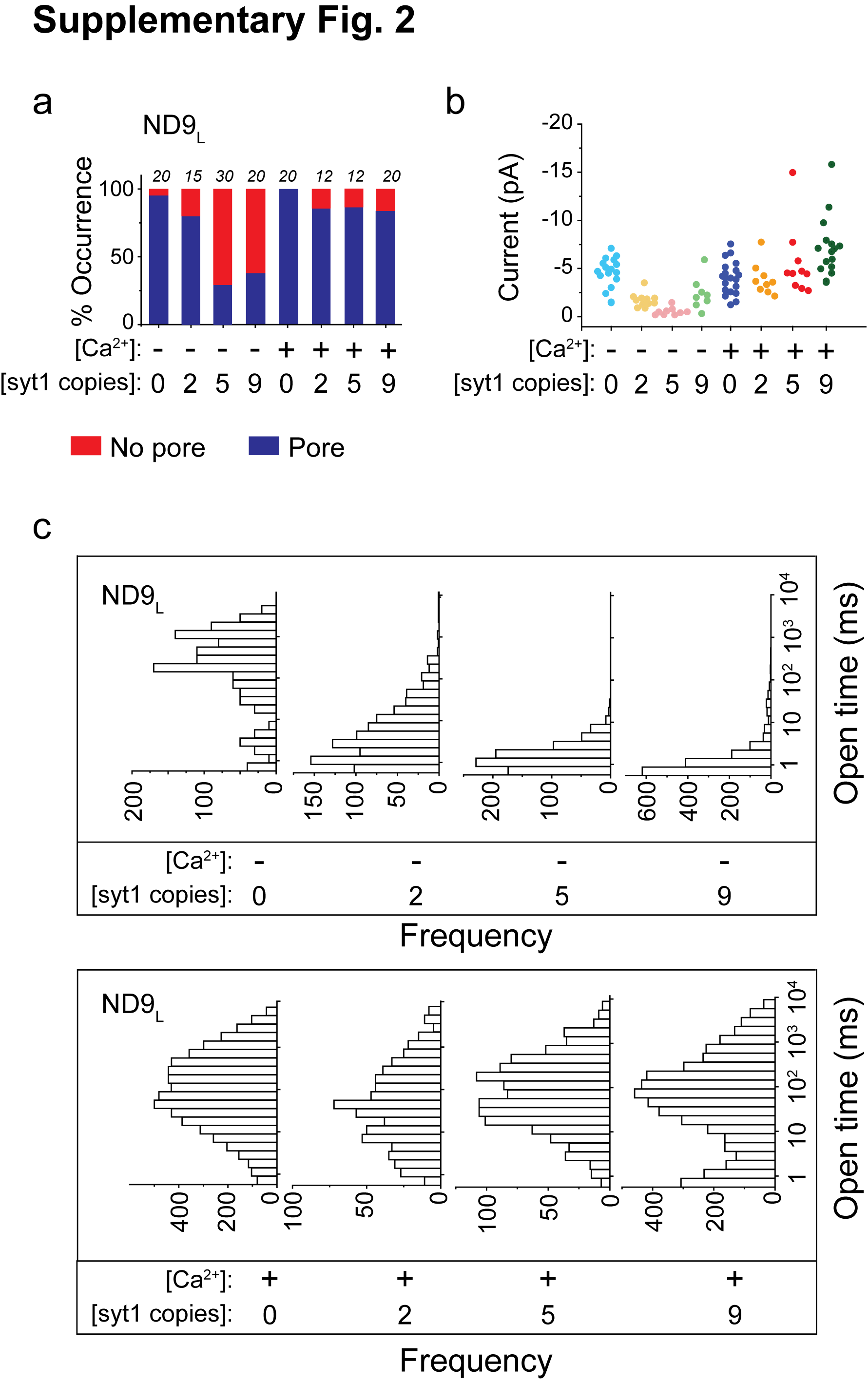


**Supplementary Fig.2. Titrating the syt1 copy number in NDs.**

**a,** Fraction of trials in which a fusion pore was detected, plotted as % occurrence: ND9_L_, in absence (-) and presence (+) of Ca^2+^, and in the absence (-) and presence (+) of the indicated number of reconstituted syt1 molecules, were compared. The total number of measurements obtained under each condition (*n*) are indicated. **b, c,** Plots of pore currents and open-time histograms, respectively, as a function of syt1 copy number.


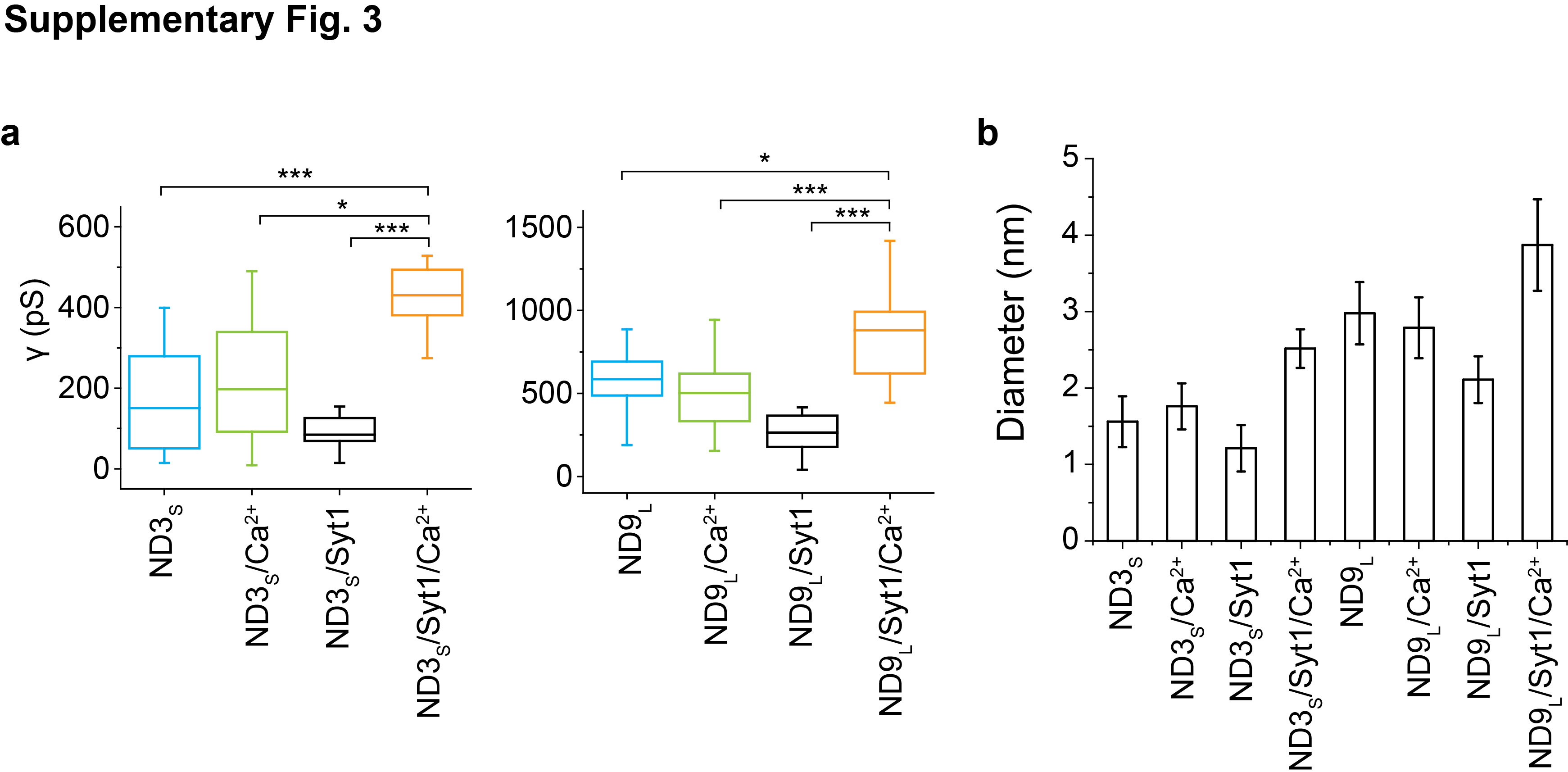


**Supplementary Fig.3. Conductance values and pore size estimates under different conditions.**

**a,** Conductance values for fusion pores formed under different conditions. Kruskal-Wallis U-test was performed, p < 0.001. Dunn’s multiple comparison post hoc test was performed thereafter, ****p* < 0.001, **p* < 0.05, (95% confidence interval). **b,** Pore diameters, calculated from the conductance values, are plotted for each indicated condition. Error bars represent SEM.


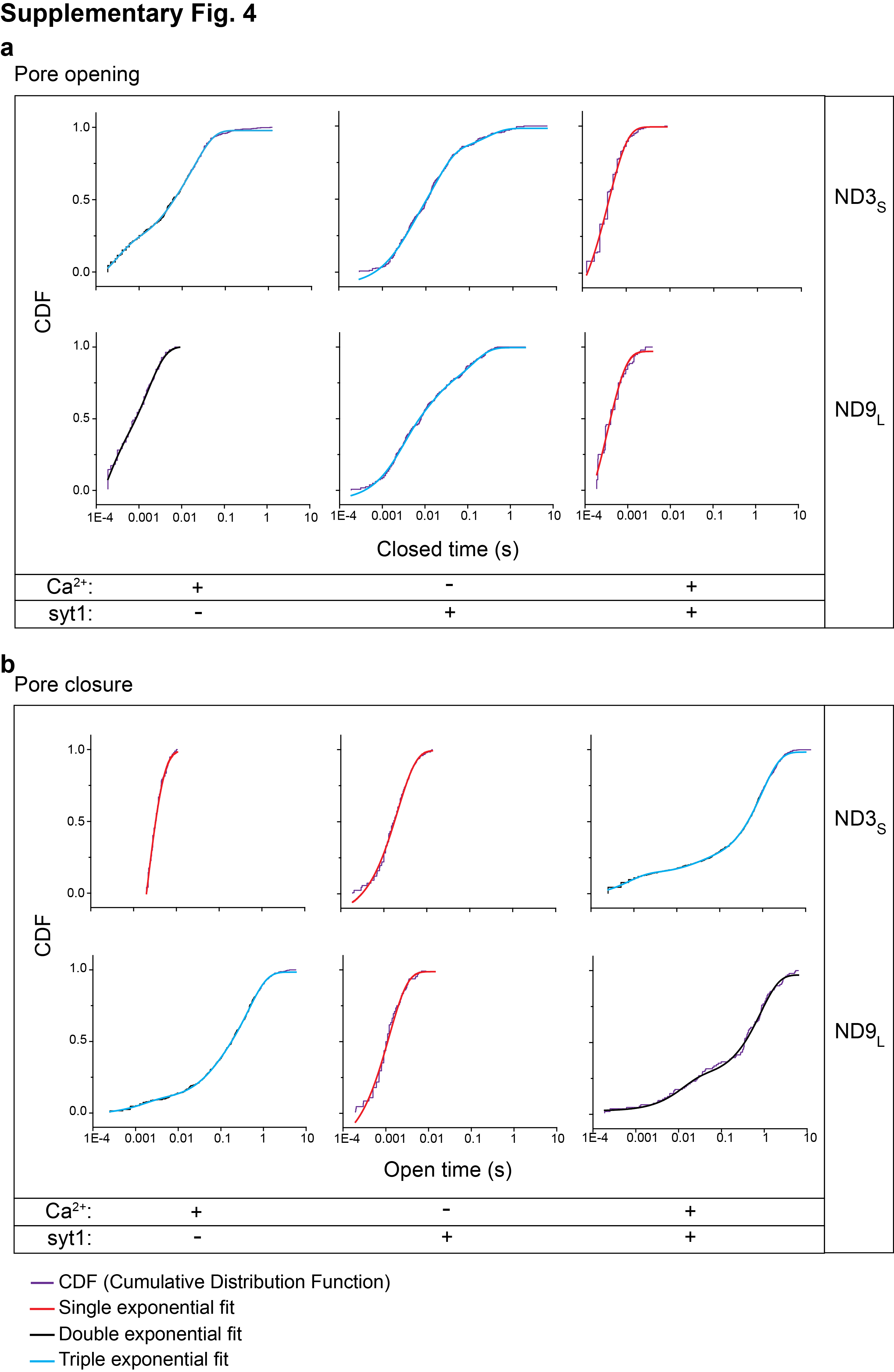


**Supplementary Fig.4. Exponential fitting of CDFs for fusion pore opening and closure.**

CDFs for pore opening **(a)** and pore closure **(b)**, under the indicated experimental conditions. Due to space limitations, only single representative examples are shown. CDFs were fitted with the indicated number of exponential functions.


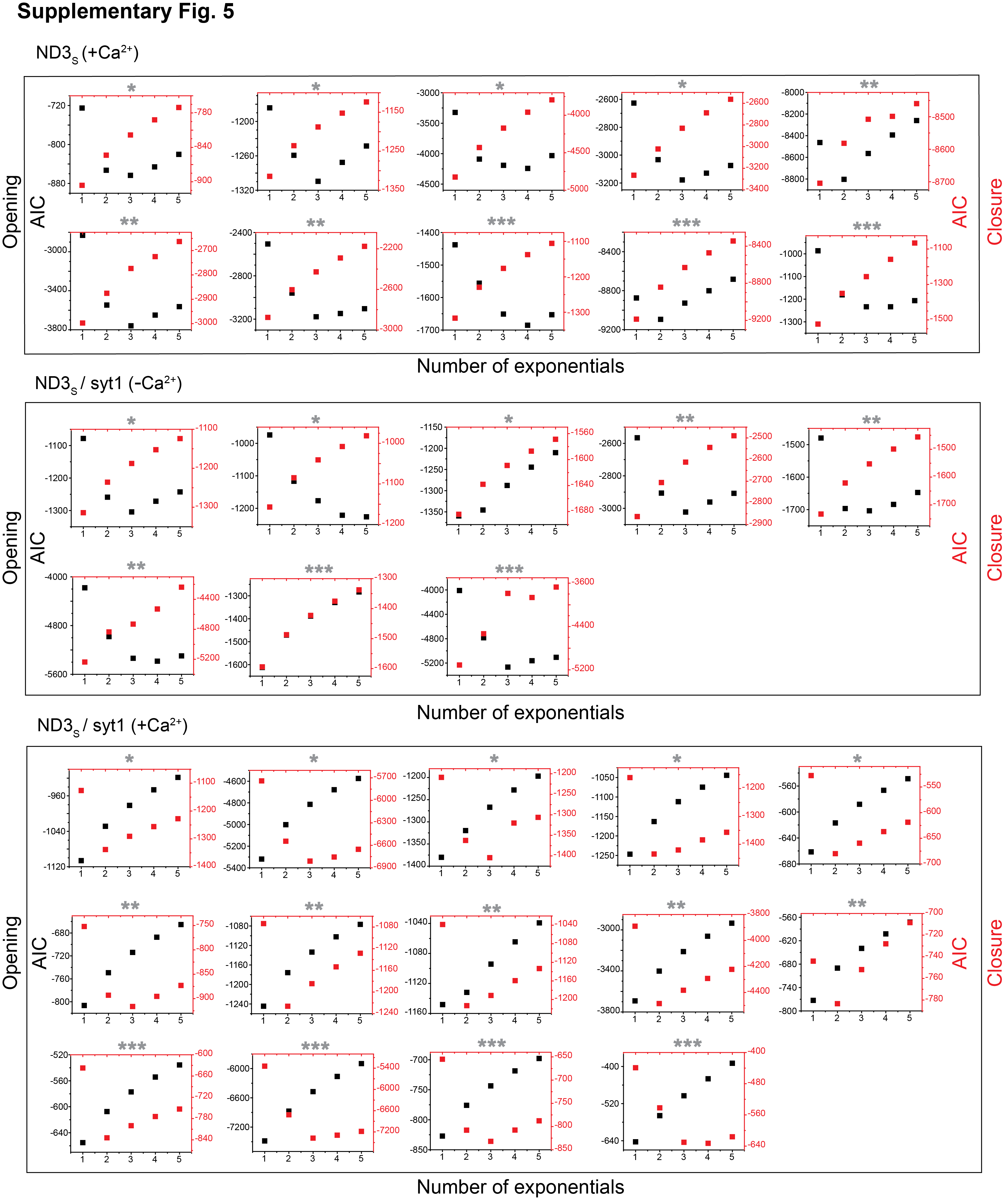


**Supplementary Fig.5. AIC plots for exponential fitting of pores formed by ND3_S_/syt1.**

To evaluate the quality of the fits to the closed (C) and open (O) state CDFs (Supplementary fig. 4; see Methods), the AIC values (Y-axis) were plotted against the number of exponentials used for fitting (X-axis), for each independent experimental trial. Opening and closure are indicated by black and red, respectively. Three sets of NDs were used; each set is demarcated with single (*), double (**) and triple (***) asterisks.


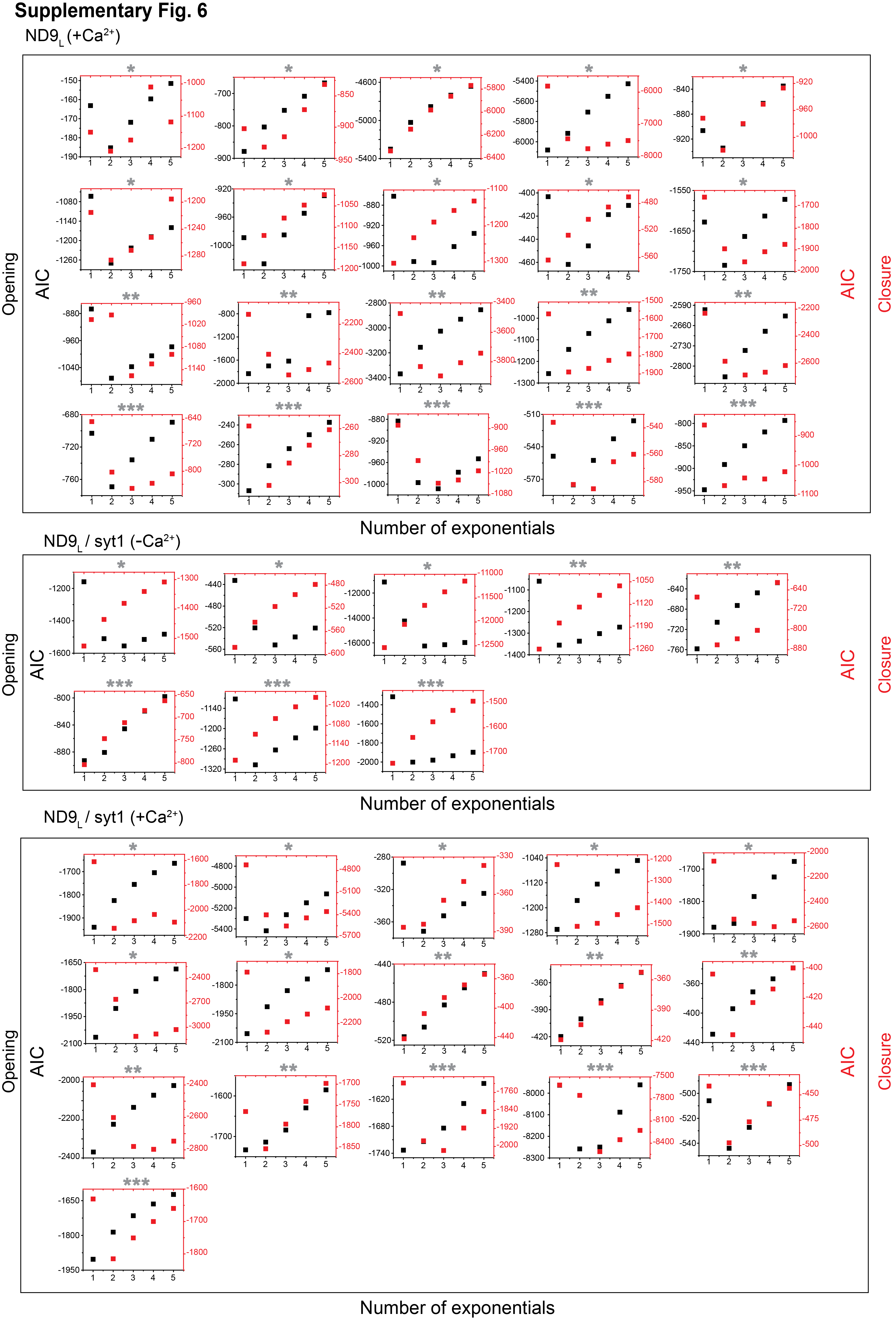


**Supplementary Fig.6. AIC plots for exponential fitting of pores formed by ND9_L_/syt1.**

Same as for Supplementary Fig.5, but using ND9_L_/syt1.


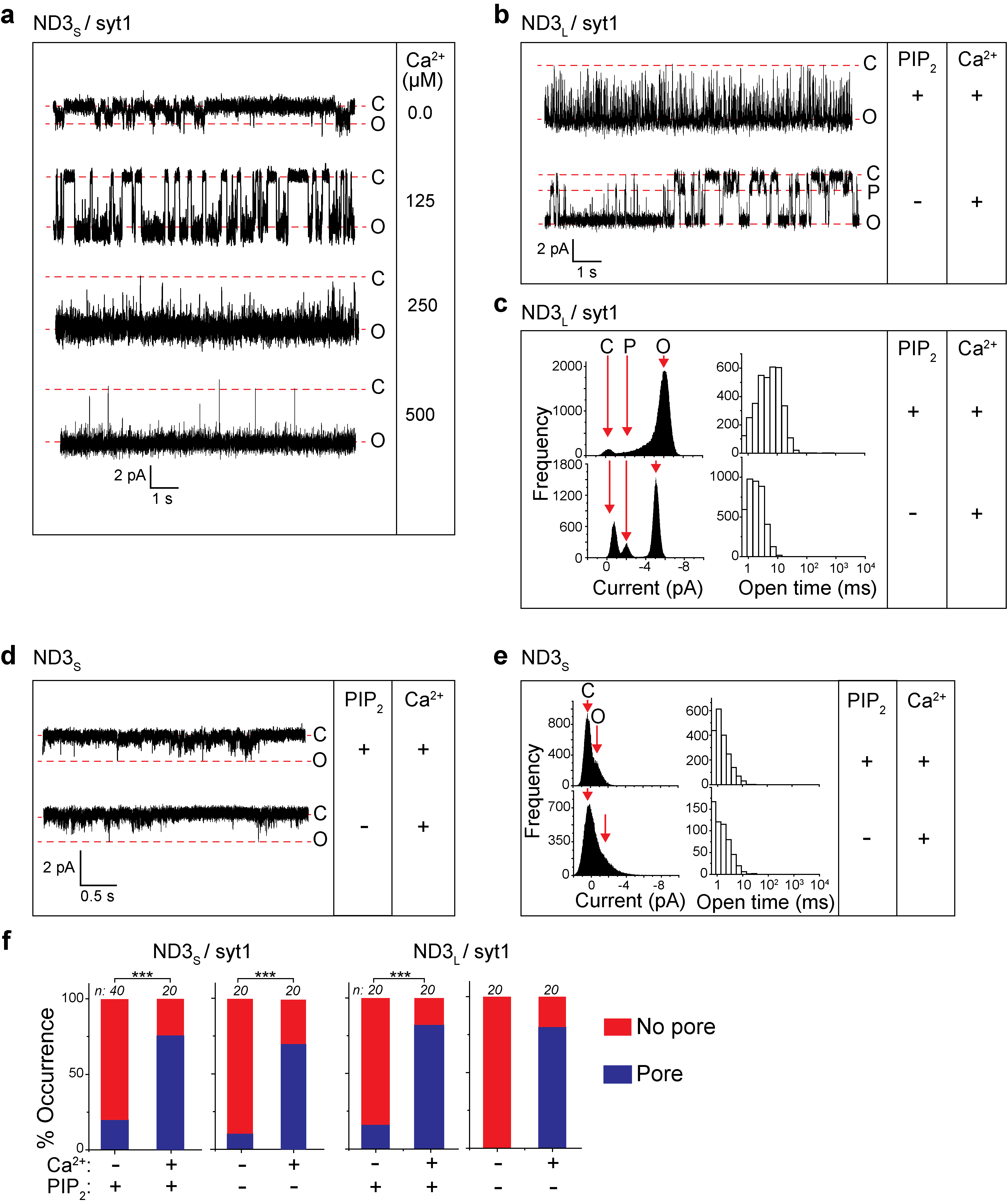


**Supplementary Fig.7. Effect of Ca^2+^ on ND3_S_/syt1 and ND3_L_/syt1 pores, and impact of PIP_2_ on ND3_S_ and ND3_L_ pores.**

**a,** Representative traces of ND3_S_/syt1 at the indicated [Ca^2+^]_free_; closed (C) and open (O) states are indicated. n = 16, 10, 10 and 15 for 0, 125, 250 and 500 μM [Ca^2+^]_free_ conditions, respectively; three independent sets of NDs were used. **b,** Representative traces of ND3_L_/syt1 in 500 μM [Ca^2+^]_free_, with and without PIP_2_ in the BLM. Closed (C), open (O), and partially open (P) states are indicated. **c,** Current and open dwell time histograms of pores are shown, for the conditions described in panel (b). **d,** Representative traces of ND3_S_ in 500 μM [Ca^2+^]_free_, with and without PIP_2_ in the BLM. **e,** Current and open dwell time histograms of pores are shown, for the conditions described in panel (d). **f,** Fraction of trials in which a fusion pore was detected, plotted as % occurrence, for ND3_S_/syt1 and ND3_L_/syt1 in 500 μM [Ca^2+^]_free_. BLMs with and without PIP_2_ were compared. Three independent sets of NDs of each type were used; n is provided above each bar. Pearson’s χ^2^ tests were performed; *** p < 0.001.


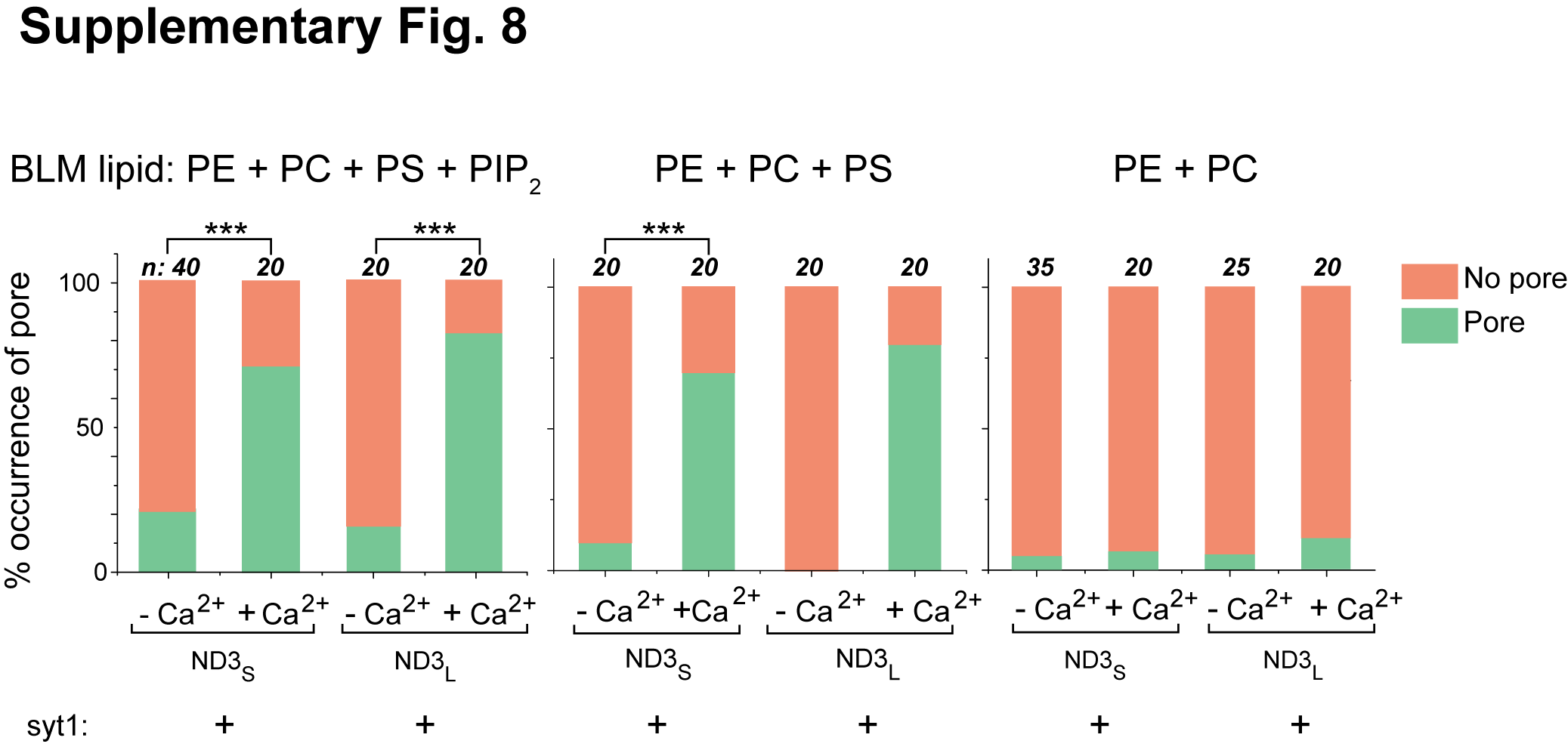


**Supplementary Fig. 8. Ca^2+^●syt1 is unable to regulate fusion pore formation**

**in the absence of PS in the BLM.**

Fraction of trials in which a fusion pore was detected, plotted as % occurrence, using ND3_S_/syt1 and ND3_L_/syt1 in the presence (+) or absence (-) of Ca^2+^, as a function of the indicated lipid composition. Three independent sets of NDs of each type were used for left and middle panel data sets, four independent sets were used for the right panel, and the total number of measurements obtained under each condition (n) is indicated. Pearson’s χ^2^ analysis was performed, ***p<0.001.


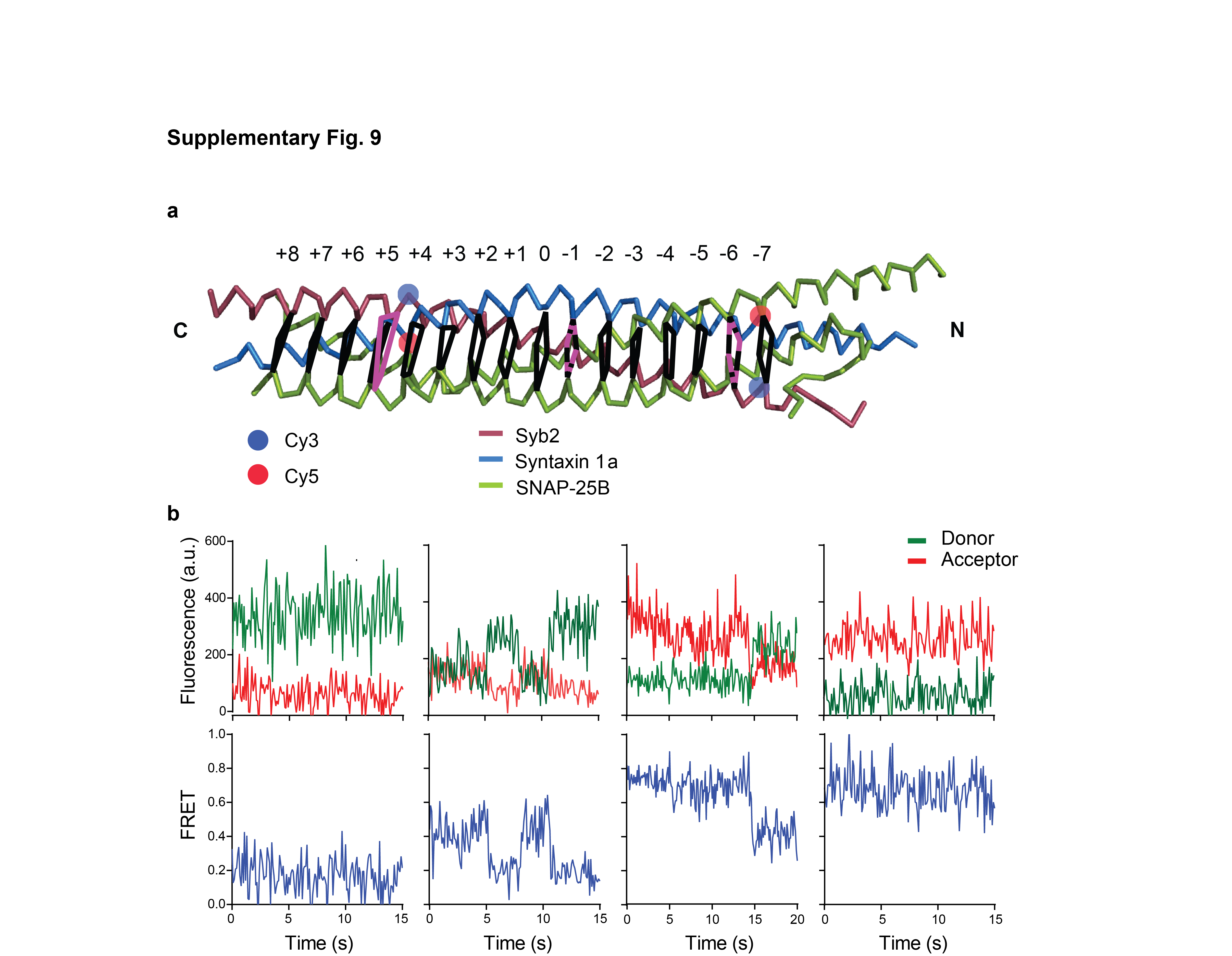


**Supplementary Fig. 9. Illustration of SNARE motifs showing positions of smFRET labels, and representative traces for the smFRET experiments in Fig.4.**

**a,** Structure of the SNARE motif four-helix bundle showing the positions of the smFRET labels used in Fig.4; cy3 and cy5 are indicated with blue and red filled circles, respectively. Layers within the SNARE motifs are indicated by numbers and are shown within the SNARE complex as black polygons. The SNARE zippering states that were identified using force measurements[^1-3^](#_ENREF_1) are shown in magenta; where these coincide with layers in the SNARE complex, the polygon is rendered with alternating black and magenta stripes. **b,** Four representative traces, obtained using the CC FRET pair in the presence of syt1 and Ca^2+^, are shown. Under this condition, all three FRET states were observed, corresponding to low (0.2), medium transition to low (0.6 to 0.2), high transition to medium (0.8 to 0.6), and high (0.8) states.


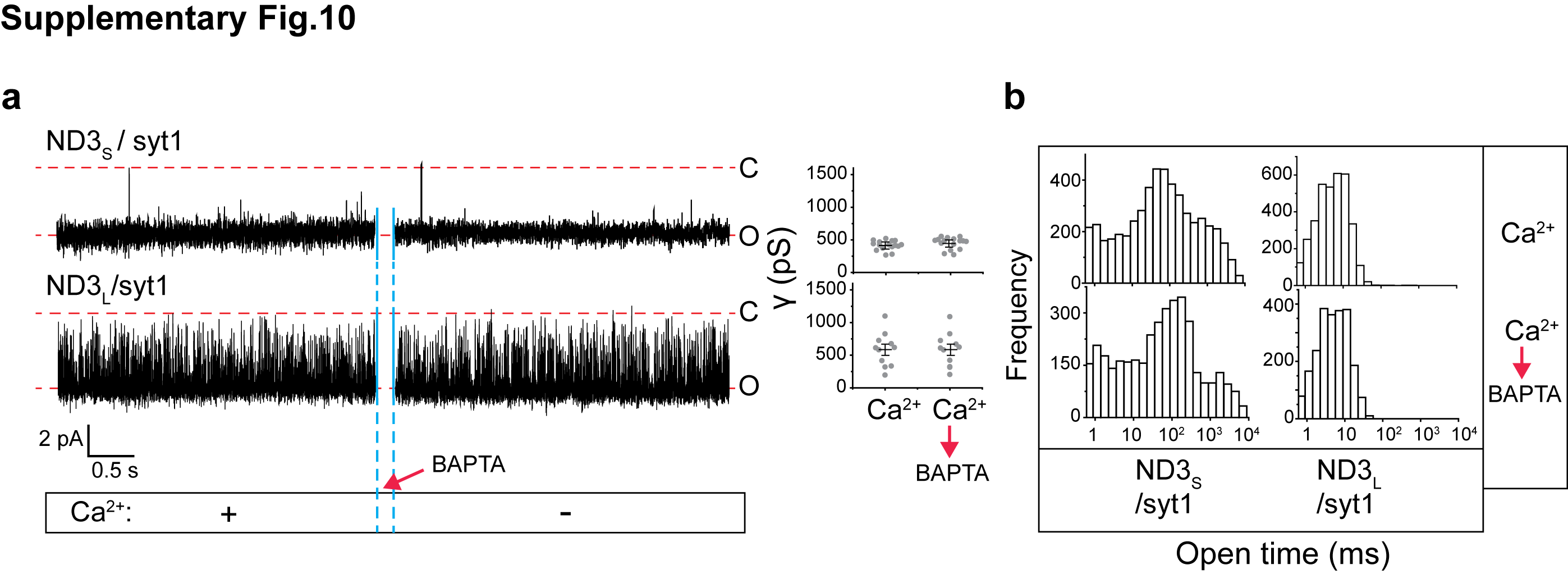


**Supplementary Fig. 10. Effect of Ca^2+^ on ND3_S_/syt1, ND3_L_/syt1 pores.**

**a,** Left panel: representative traces of ND3_S_/syt1 and ND3_L_/syt1. After pores were opened with 500 μM [Ca^2+^]_free_, 1500 μM excess BAPTA was added. The current/time scale, for all traces, is shown on the left. Right panel: pore conductance values were plotted. Error bars indicate SEM; n = 16 and 11, and three independent sets of NDs were used. **b,** Open dwell time histograms, from the same recordings analyzed in panel a, were plotted.


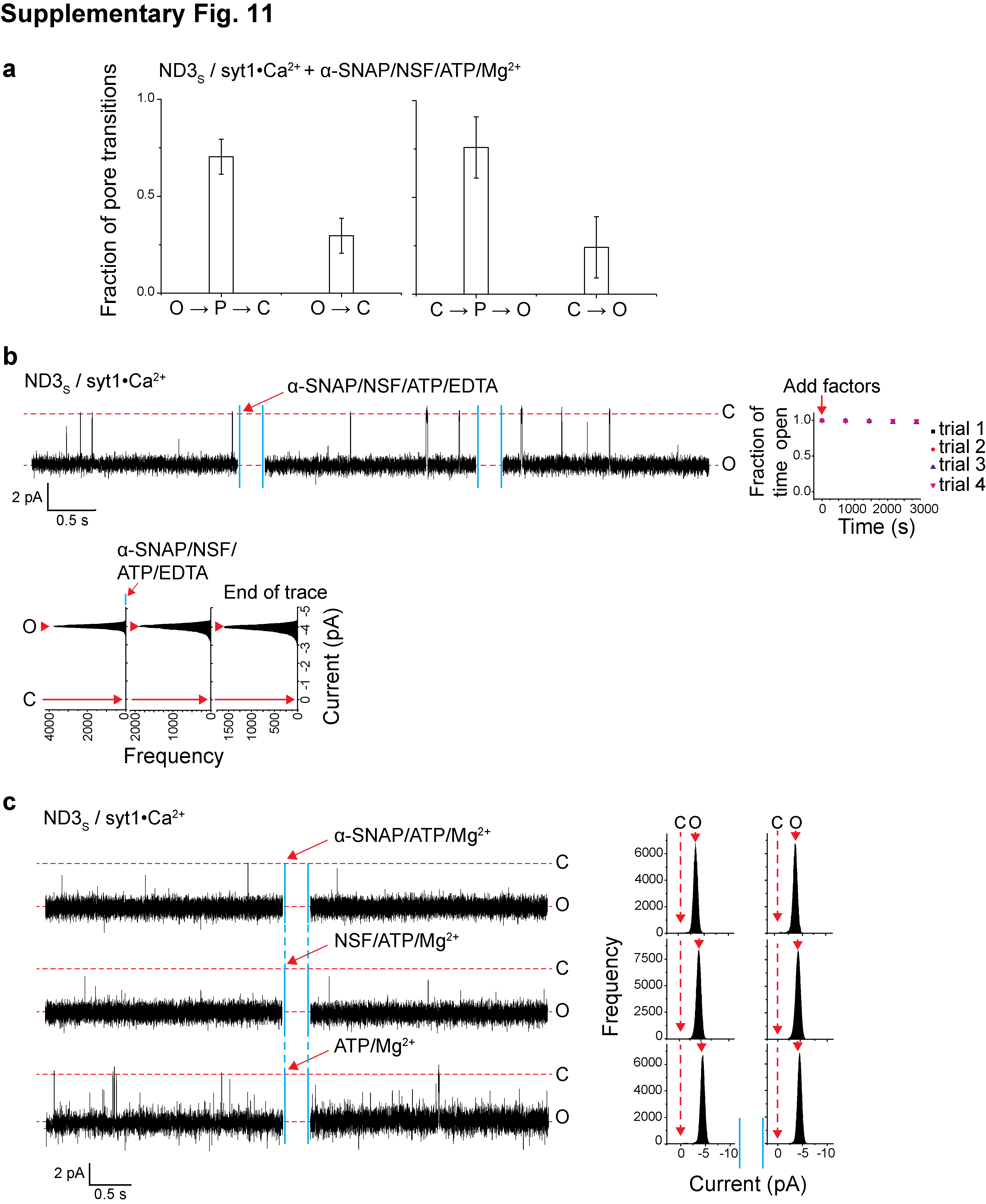


**Supplementary Fig. 11. α-SNAP, NSF, and ATP are all required to close fusion pores that were triggered to open by Ca^2+^•syt1.**

**a,** Fraction of open-to-closed, and closed-to-open transitions that were and were not preceded (during closure), or followed (during opening), by a partial sub-conductance state (P). Error bar represents SEM. **b,** Representative traces of ND3_S_/syt1 pores at 500 μM [Ca^2+^]_free_. After pore formation, α-SNAP, NSF, ATP, EDTA were added (red arrow). Closed (C) and open (O) states are marked; the current/time scales are shown below the traces. *Right panel:* after the addition of factors in **(b)**, recordings were divided arbitrarily into five 720 second epochs; the fraction of time the pores were open during each epoch was quantified for individual traces and plotted. Current histograms, corresponding to each of the three epochs in **(b)** are shown beneath the trace. **c,** Representative traces of ND3_S_/syt1 pores at 500 μM [Ca^2+^]_free_; following pore formation, different combinations of α-SNAP, NSF, ATP, EDTA were added (red arrow). Closed (C) and open (O) states are marked; the current/time scales are shown below the traces. Current histograms, corresponding to each of the epochs in panel **(c)** are shown beside each trace. Number of independent BLMs: 4 in panel **(b)**; 3, 4 and 3, from top to bottom, in panel **(c)**; two different sets of NDs used.

**
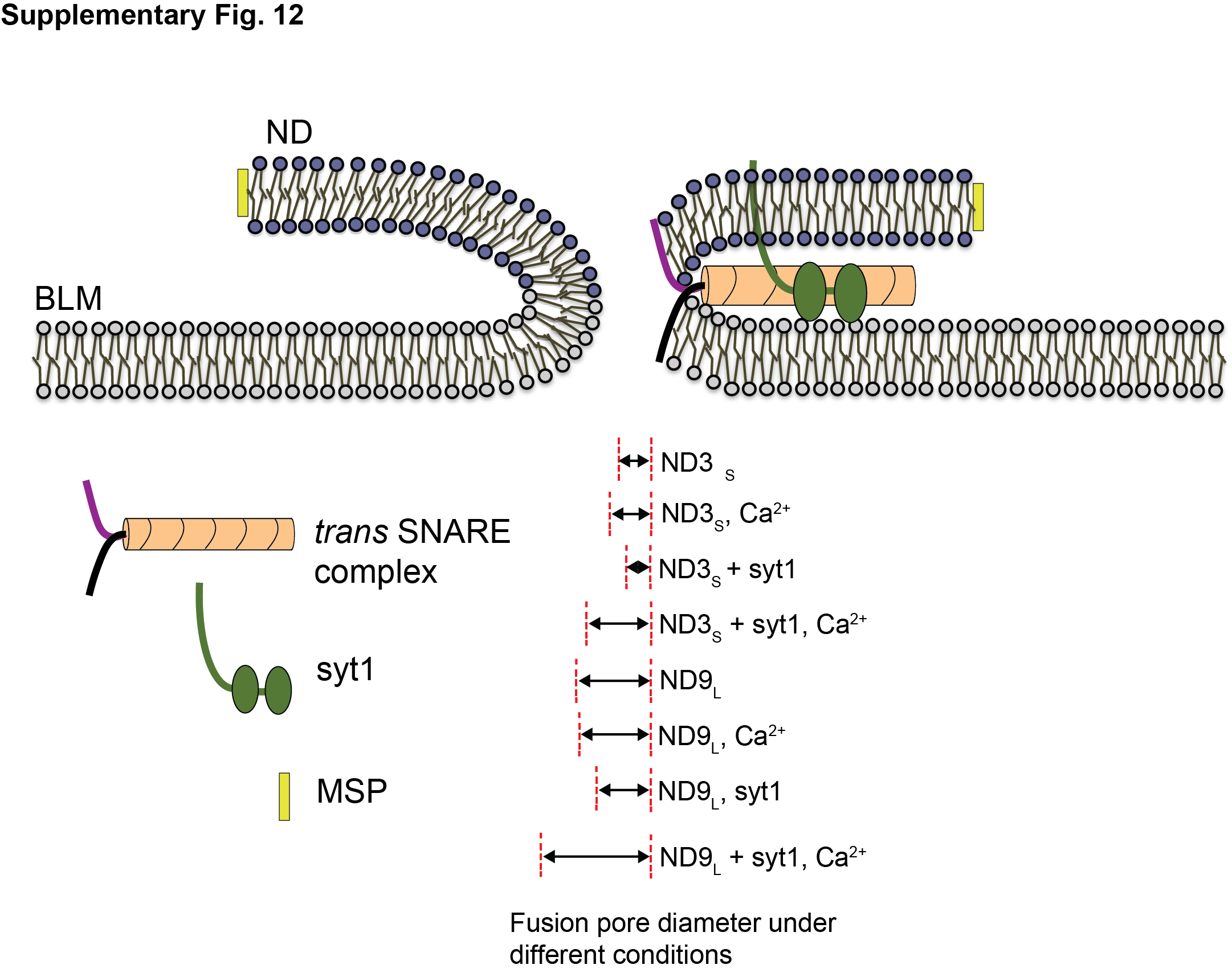
**

**Supplementary Fig. 12.** Scale drawing of the fusion pore components used in the current study, along with the pore size estimates, under the indicated conditions, as estimated from conductance measurements, shown in the Supplementary Fig. 3. The black and purple lines indicate the TMDs of syntaxin 1a and syb2, respectively. For clarity, the four-helix bundle that comprises the core of the *trans*-SNARE complex is shown as a cylinder, and the model reflects a hybrid fusion pore formed by both lipids and the TMDs of SNARE proteins[^4^](#_ENREF_4).


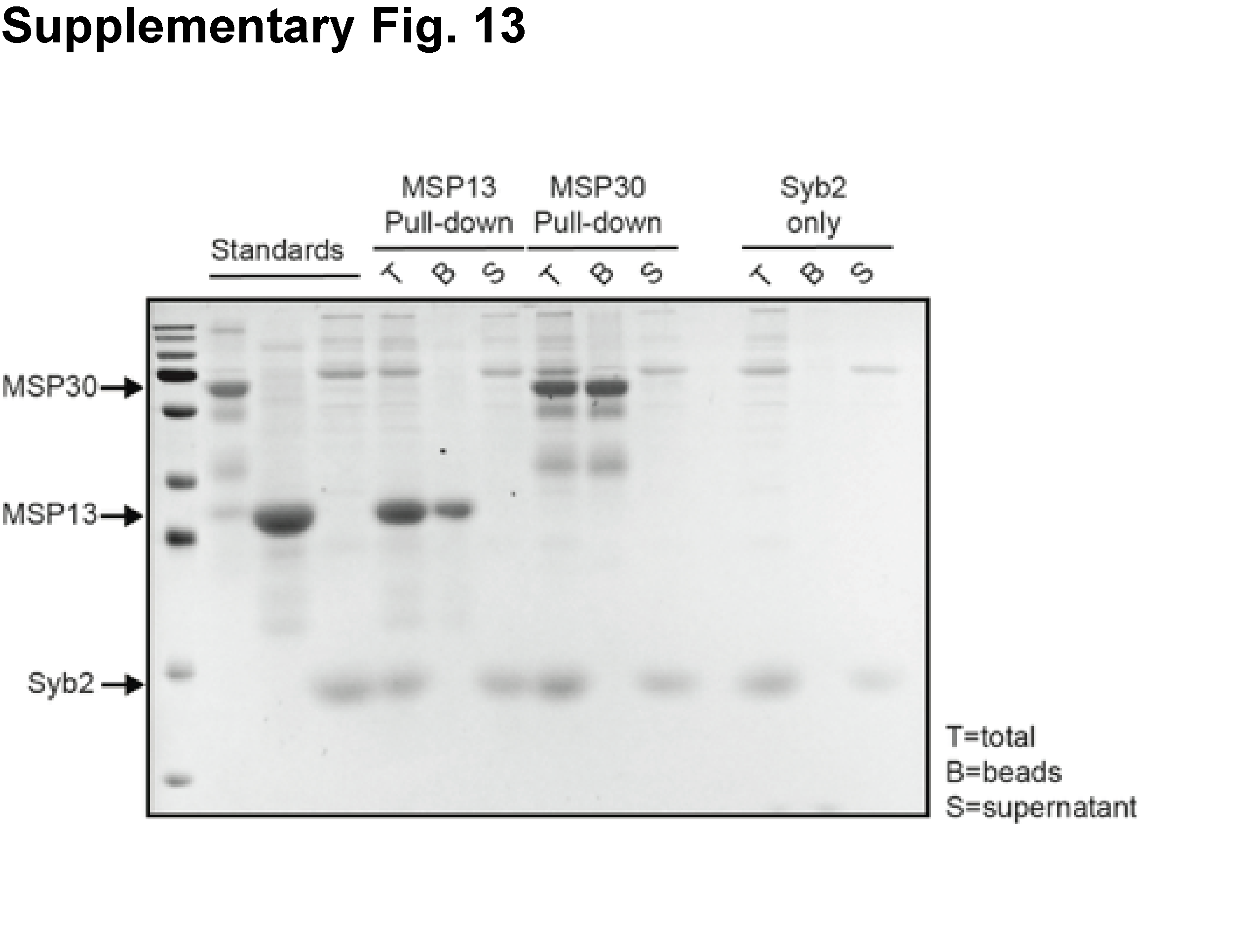


**Supplementary Fig. 13. Immobilized MSP13 and MSP30 fail to pull-down syb2.**

Pull-down assays were carried out by first incubating Ni-beads with his_6_-MSP13 or his_6_-MSP30; free MSP was removed by washing 3x with 50 mM imidazole. Purified full-length syb2 was then incubated with bead-bound MSP in detergent (1% octylglucoside) at 4ºC. Beads were washed three times in binding buffer (25 mM HEPES (pH: 7.5), 100 mM KCl and 1% OG) containing 50 mM imidazole. Three samples from each assay were subjected to SDS-PAGE: total (T; input), beads (B; bead-bound material after washing), and supernatant (S; unbound material that was removed before washing). In each case, 10% of the samples were loaded, and proteins were visualized by staining with Coomassie blue. From the gel, it is apparent that syb2 does not interact with MSP under these conditions.

**References**

1. Gao, Y. et al. Single reconstituted neuronal SNARE complexes zipper in three distinct stages. *Science (New York, N Y )* **337**, 1340-3 (2012).

2. Zorman, S. et al. Common intermediates and kinetics, but different energetics, in the assembly of SNARE proteins. *eLife* **3**, e03348 (2014).

3. Ma, L. et al. Munc18-1-regulated stage-wise SNARE assembly underlying synaptic exocytosis. *eLife* **4**(2015).

4. Bao, H. et al. Exocytotic fusion pores are composed of both lipids and proteins. *Nat Struct Mol Biol* **23**, 67-73 (2016).
